# Neurons secrete *miR-132*-containing exosomes to regulate brain vascular integrity

**DOI:** 10.1038/cr.2017.62

**Published:** 2017-04-21

**Authors:** Bing Xu, Yu Zhang, Xu-Fei Du, Jia Li, Hua-Xing Zi, Ji-Wen Bu, Yong Yan, Hua Han, Jiu-Lin Du

**Affiliations:** 1 Institute of Neuroscience, State Key Laboratory of Neuroscience, Center for Excellence in Brain Science and Intelligence Technology, Chinese Academy of Sciences, 320 Yue-Yang Road, Shanghai 200031, China; 2 University of Chinese Academy of Sciences, 19A Yu-Quan Road, Beijing 100049, China; 3 School of Life Science and Technology, ShanghaiTech University, 319 Yue-Yang Road, Shanghai 200031, China; 4 Institute of Automation, Center for Excellence in Brain Science and Intelligence Technology, Chinese Academy of Sciences, 95 Zhong-guan-cun East Road, Beijing 100190, China

**Keywords:** brain vascular integrity, neuron, endothelial cell, exosome, *miR-132*, *eef2k*, zebrafish

## Abstract

Vascular integrity helps maintain brain microenvironment homeostasis, which is critical for the normal development and function of the central nervous system. It is known that neural cells can regulate brain vascular integrity. However, due to the high complexity of neurovascular interactions involved, understanding of the neural regulation of brain vascular integrity is still rudimentary. Using intact zebrafish larvae and cultured rodent brain cells, we find that neurons transfer *miR-132*, a highly conserved and neuron-enriched microRNA, via secreting exosomes to endothelial cells (ECs) to maintain brain vascular integrity. Following translocation to ECs through exosome internalization, *miR-132* regulates the expression of vascular endothelial cadherin (VE-cadherin), an important adherens junction protein, by directly targeting *eukaryotic elongation factor* *2* *kinase* (*eef2k*). Disruption of neuronal *miR-132* expression or exosome secretion, or overexpression of vascular *eef2k* impairs VE-cadherin expression and brain vascular integrity. Our study indicates that *miR-132* acts as an intercellular signal mediating neural regulation of the brain vascular integrity and suggests that the neuronal exosome is a novel avenue for neurovascular communication.

## Introduction

Brain vascular endothelial cells (ECs) connect with each other through junction proteins and are enveloped by surrounding pericytes and astrocytes to form the blood-brain barrier (BBB)^1,2,3,4^. The BBB restricts material exchange between the brain and blood vessels to maintain the homeostasis of the brain microenvironment, which is crucial for the normal development and function of the central nervous system (CNS)^1,2,3,4^. It is generally believed that the development of brain vasculature, including its integrity, is tightly regulated by surrounding neural tissues. In the past several years, several signaling pathways, in particular the Wnt and Sonic Hedgehog (Shh) pathways, were found to be implicated in this neural regulation^4,5^. During embryogenesis, neural progenitors secrete Wnt ligands, which activate canonical Wnt signaling in ECs and contribute to the maturation of brain vasculature^6,7,8^. At postnatal stages, astrocyte-secreted Shh further promotes vascular integrity of the brain by activating Hedgehog signaling in ECs through the receptor Patched-1^9^. However, the understanding of the neural regulation of brain vascular integrity is still rudimentary.

Exosomes are small membrane vesicles that originate from the inward budding of endosomal membranes^10^. Diverse types of cells including neurons can secrete exosomes, which transfer functional proteins^11,12^, mRNAs and/or microRNAs (miRNAs)^13,14^ to recipient cells. Exosome-mediated material transfer has been recognized as an important route for intercellular communication^15^. Recent studies show that neurons can secrete exosomes to regulate neural development^16,17^. For example, motor neuron terminals in *Drosophila* larvae release exosomes to promote the maturation of neuromuscular junctions^11,18^. As blood vessels in the developing brain are sparsely covered by mural cells, it is possible that neuronal exosomes can be directly taken up by adjacent ECs to mediate the neural regulation of brain vascular development.

In the present study, we found that, neurons transfer *miR-132*, an evolutionarily conserved and neuron-enriched miRNA^19,20^, into ECs via secreting exosomes to regulate the brain vascular integrity. Knockdown or mutation of *miR-132* caused severe intracranial hemorrhage and disruption of brain vascular integrity in zebrafish larvae with reduced expression of the adherens junction protein vascular endothelial cadherin (VE-cadherin, also known as Cdh5) and its intracellular partner β-catenin. The defect in the brain vascular integrity was mimicked by neuron-specific reduction of *miR-132*. Interestingly, up- and downregulation of the *miR-132* level in neurons resulted in a coordinated change of the *miR-132* level in ECs, and EC-specific reduction of *miR-132* also impaired Cdh5 expression as well as the brain vascular integrity. Combining experiments on cultured rodent brain cells, we found that neurons secreted *miR-132*-containing exosomes, which were then internalized into brain ECs, leading to an increased level of *miR-132* in ECs, and impairment of exosome secretion in zebrafish larvae caused intracranial hemorrhage. Furthermore, we identified *eukaryotic elongation factor 2 kinase* (*eef2k*) as a direct target of *miR-132*. Gain- and loss-of-function experiments showed that *eef2k* in ECs mediated the action of *miR-132* on Cdh5 expression and brain vascular integrity. This study discovers a previously unidentified function of *miR-132* and reveals that neuronal exosomes serve as a novel carrier in mediating the neural regulation of brain vascular integrity.

## Results

### *MiR-132* is necessary for the brain vascular integrity in larval zebrafish

MiRNAs are small non-coding RNAs that post-transcriptionally regulate the expression of target mRNAs^21^. *MiR-132* is a neuron-enriched miRNA and plays crucial roles in neural development and plasticity^19,20^. *MiR-132* is evolutionarily conserved among animal species and highly expressed in the brain of zebrafish larvae (Supplementary information, Figure S1A and S1B; see also ref.^22^).

**Figure 1:**
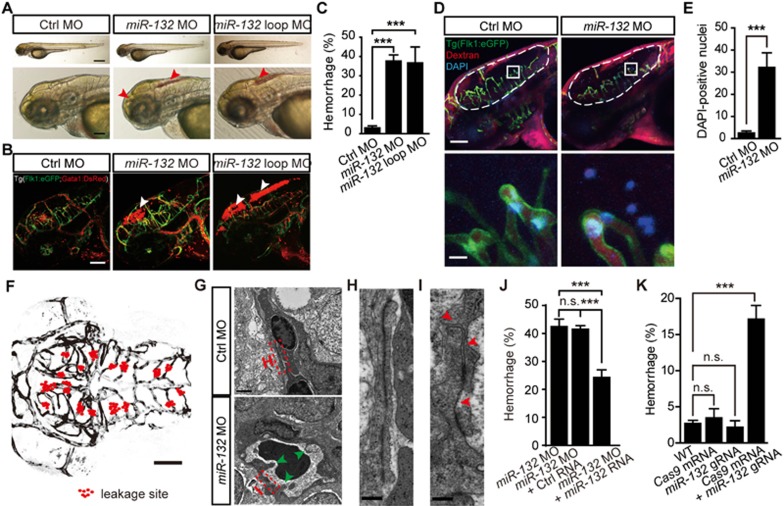
*MiR-132* knockdown and mutation impair brain vascular integrity of larval zebrafish. **(A**, **B)** Representative images showing that *miR-132* knockdown with morpholino oligonucleotides (MOs) caused intracranial hemorrhage (arrowheads) in wildtype larvae **(A)** and blood cell leakage (arrowheads) in the brain of Tg(Flk1:eGFP;Gata1:DsRed) larvae **(B)**. The images were taken from larvae at 3 days post fertilization (dpf). **(C)** Summary of the intracranial hemorrhage effect of *miR-132* knockdown. The experiments were repeated 4-8 times, and at each time > 89 embryos were examined for each group. **(D**, **E)** Representative images and summary showing that *miR-132* knockdown caused more DAPI-positive brain parenchymal nuclei (blue) in 3-dpf Tg(Flk1:eGFP) larvae, in which a mixture of DAPI and Alex-568 dextran were injected into the blood circulation system. In **D**, the area outlined by the rectangles in the top are enlarged in the bottom, and the number of DAPI-positive nuclei in the area outlined by the dashed lines were counted. 13 control embryos and 18 morphants were analyzed in **E**. **(F)** Spatial distribution of leakage sites (red dots) in all 9 *miR-132* morphants examined by *in vivo* time-lapse confocal imaging. **(G-I)** Transmission electron microscopy (TEM) images showing the ultrastructural changes of brain vessels in *miR-132* morphants. The areas outlined by the red dashed rectangles in **G** were enlarged in (**H**, “Ctrl MO”) and (**I**, “*miR-132* MO”). The green arrowheads in **G** point to the abnormal sites of the vessel luminal side, and the red arrowheads in **I** point to the abnormal junctions between vascular endothelial cells (ECs). **(J)** Rescue effect of *miR-132*RNA overexpression on intracranial hemorrhage defects in *miR-132* morphants. The experiments were repeated four times, and at each time > 75 embryos were examined for each group. **(K)** Summary of the intracranial hemorrhage effect of *miR-132* mutations generated by co-injection of zebrafish codon-optimized *Cas9* mRNA and *miR-132* gRNA. The experiments were repeated 3-5 times, and for each time > 21 embryos were examined for each group. Scale bar, 400 μm (top) and 100 μm (bottom) **(A)**, 100 μm **(B)**, 100 μm (top) and 10 μm (bottom) **(D)**, 100 μm **(F)**, 1 μm **(G)**, 200 nm **(H** and **I)**. Error bars, SEM. n.s., no significant; ^***^P < 0.001 (one-way ANOVA with *post hoc* Bonferroni's multiple comparison test for **C** and **K**; unpaired two-tailed Student's *t*-test for **E**; one-way ANOVA with *post hoc* Tukey's multiple comparison test for **J**).

To examine whether *miR-132* is important for brain vascular development, we downregulated the expression of *miR-132* using two morpholino oligonucleotides (MOs), which targeted mature *miR-132* (“*miR-132* MO”, see also ref.^23^) or precursor *miR-132* loop (“*miR-132* loop MO”, Supplementary information, Figure S1C). The expression of *miR-132* in zebrafish larvae was efficiently downregulated by these MOs (Supplementary information, Figure S1D; *P* < 0.05). In comparison with embryos injected with a control MO (“Ctrl MO”), a substantial proportion of *miR-132* morphants displayed intracranial hemorrhage (Figure 1A and 1C; Ctrl MO: 3.3% ± 0.7%; *miR-132* MO: 38.0% ± 2.8%; *miR-132* loop MO: 37.1% ± 7.9%; mean ± SEM, *P* < 0.001) without any marked embryonic death or defects in gross morphology (Supplementary information, Figure S1E and S1F). To demonstrate the leakage of blood cells in the brain, we knocked down *miR-132* expression in double transgenic zebrafish Tg(Flk1:eGFP;Gata1:DsRed) larvae, in which ECs express enhanced green fluorescent protein (eGFP) and blood cells express DsRed. This allowed us to observe the accumulation of DsRed-expressing blood cells in brain ventricles (Figure 1B). Blood cell leakage in brain ventricles was further confirmed by transmission electron microscopy (TEM) (Supplementary information, Figure S1G).

Next, we investigated whether the intracranial hemorrhage induced by *miR-132* knockdown was due to the impairment of brain vascular integrity. To this end, we first injected a solution containing DAPI and Alex-568 dextran (10 kDa molecular mass) into the blood circulation system through the common cardinal vein in Tg(Flk1:eGFP) larvae 3 days post fertilization (dpf) and evaluated the degree of vascular leakage by counting DAPI-positive nuclei of brain parenchymal cells 30 min later^24^. A significant increase of DAPI-positive parenchymal cell nuclei as well as dextran leakage was observed in *miR-132* morphants (Figure 1D, 1E and Supplementary information, Figure S1H; *P* < 0.001). Second, we injected red fluorescent beads (100 nm in diameter) into the blood circulation system of Tg(Flk1:eGFP) larvae and immediately monitored bead leakage by *in vivo* time-lapse confocal imaging. In contrast to control embryos, red fluorescent beads were readily observed to leak out from the brain vessels of *miR-132* morphants (Supplementary information, Figure S1I and S1J). More importantly, 17 out of 18 leakage events observed occurred at functional vessels which already exhibited blood flow (Figure 1F and Supplementary information, Figure S1J; data obtained from 9 *miR-132* morphants). This suggested that the vascular leakage in brains of *miR-132* morphants is due to defects in the integrity of brain vasculature. Third, we examined the ultra-structure of brain vessels using TEM. EC-EC contacts in control larvae showed numerous and extensive junctions (Figure 1G and 1H) whereas junctions between brain ECs in hemorrhagic *miR-132* morphants was discontinuous, junction density was reduced, and the cleft between ECs was increased (Figure 1G and 1I). The luminal side of brain vessels in *miR-132* morphants was tortuous and rough (Figure 1G). Consistent with a previous finding that *miR-132* can facilitate pathological angiogenesis^25^, we also found the brain vascular density was decreased in *miR-132* morphants (Supplementary information, Figure S2; *P* < 0.01).

Because only about 40% of *miR-132* morphants exhibited severe intracranial hemorrhage (Figure 1C), we examined the level of *miR-132* in hemorrhagic and non-hemorrhagic *miR-132* morphants, and found that the expression of *miR-132* was comparable in the two groups (Supplementary information, Figure S3A). We noted, however, in those *miR-132* morphants that did not exhibit hemorrhage, brain vascular integrity was also disrupted as evidenced by a significant increase of DAPI leakage from the brain vasculature (Supplementary information, Figure S3B and S3C; *P* < 0.01).

This defect of brain vascular integrity was a specific consequence of downregulation of *miR-132*, because co-injection of *miR-132* RNA with *miR-132* MO alleviated the hemorrhagic phenotype in morphants (Figure 1J; *P* < 0.001). Moreover, *miR-132* mutations induced by co-injecting *miR-132* guide RNA (gRNA) and zebrafish codon-optimized *Cas9* mRNA also led to a significant increase of intracranial hemorrhage in F0 embryos (Figure 1K and Supplementary information, Figure S4; *P* < 0.001). Taken together, these results indicate that *miR-132* is necessary for the integrity of the brain vasculature.

### Cdh5 and its intracellular partner β-catenin are involved in brain vascular integrity

**Figure 2:**
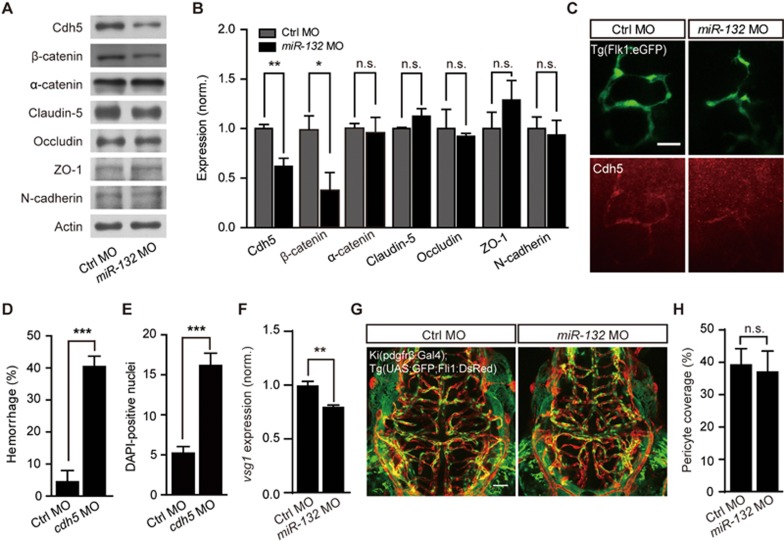
Involvement of Cdh5 in *miR-132* knockdown-induced defects of brain vascular integrity. **(A-C)** Effects of *miR-132* knockdown on the expression of junction proteins. Representative blots **(A)** and summary **(B)** of Western blot analyses. The experiments were repeated 3-8 times. **(C)** Cdh5 immunostaining in 3-dpf Tg(Flk1:eGFP) larvae. **(D** and **E)** Effects of *Cdh5* knockdown on intracranial hemorrhage **(D)** and DAPI leakage **(E)** in 3-dpf larvae. The experiments were repeated 6 times and at each time > 49 embryos were examined for each group in **D**. 23 control embryos and 24 morphants were analyzed in **E**. **(F)** Effect of *miR-132* knockdown on *vsg1* expression. The experiments were repeated three times. **(G**, **H)** Representative projected confocal images **(G)** and summary **(H)** showing no significant effect of *miR-132* knockdown on the pericyte coverage of brain blood vessels. 3-dpf larvae generated by crossing Ki(pdgfrβ:Gal4) with Tg(UAS:GFP;Fli1:DsRed) were used, and GFP and DsRed are expressed in pericytes and ECs, respectively. 6 control embryos and 6 *miR-132* morphants were analyzed. Scale bar, 25 μm **(C)**, 50 μm **(G)**. Error bars, SEM. n.s., not significant; ^*^P < 0.05, ^**^P < 0.01, ^***^P < 0.001 (unpaired two-tailed Student's *t*-test for **B**, **D**, **E**, **F** and **H**).

The maintenance of brain vascular integrity requires tight connections between ECs through junction proteins^2^. In hemorrhagic *miR-132* morphants, the expression of Cdh5, an important component of adherens junctions^26^, but not Claudin-5, Occludin, ZO-1 or N-cadherin was markedly reduced (Figure 2A-2C; *P* < 0.01). To further confirm changes in adherens junction proteins, we examined the expression of α-catenin and β-catenin, which are important intracellular partners of Cdh5^26^. The expression of β-catenin was reduced in *miR-132* morphants (*P* < 0.05), whereas we observed no significant change for α-catenin (Figure 2A and 2B). Similar to the effects of *miR-132* knockdown, *Cdh5* morphants also displayed increased intracranial hemorrhage (see also ref.^27^) and DAPI leakage (Figure 2D, 2E and Supplementary information, Figure S5; *P* < 0.001).

We then chose to examine the effect of *miR-132* knockdown on endothelial transcytosis and pericytes that can also regulate vascular permeability in the brain^4^. Zebrafish *vessel-specific 1* (*vsg1*) is an ortholog of the mammalian *plasmalemma vesicle associated protein* (*Plvap*)^28^, which is a critical mediator of endothelial transcytosis^29,30^. We found that *vsg1* mRNA was reduced rather than increased in *miR-132* morphants (Figure 2F; *P* < 0.01), suggesting that at least transcytosis is not enhanced by *miR-132* knockdown. To examine changes in coverage of brain vessels by pericyte, we first generated pdgfrβ:Gal4 knockin zebrafish, named Ki(pdgfrβ:Gal4), in which Gal4 is specifically expressed in pericytes (Materials and Methods). By crossing Ki(pdgfrβ:Gal4) with Tg(UAS:GFP;Fli1:DsRed), we generated larvae in which pericytes express GFP and ECs express DsRed. These allowed us to determine that pericyte coverage was not significantly changed in *miR-132* morphants (Figure 2G and 2H). Collectively, these results suggest that *miR-132* regulates the brain vascular integrity through affecting adherens junction proteins rather than transcytosis or pericytes.

### Neuronal *miR-132* regulates brain vascular integrity via affecting vascular *miR-132* level

**Figure 3:**
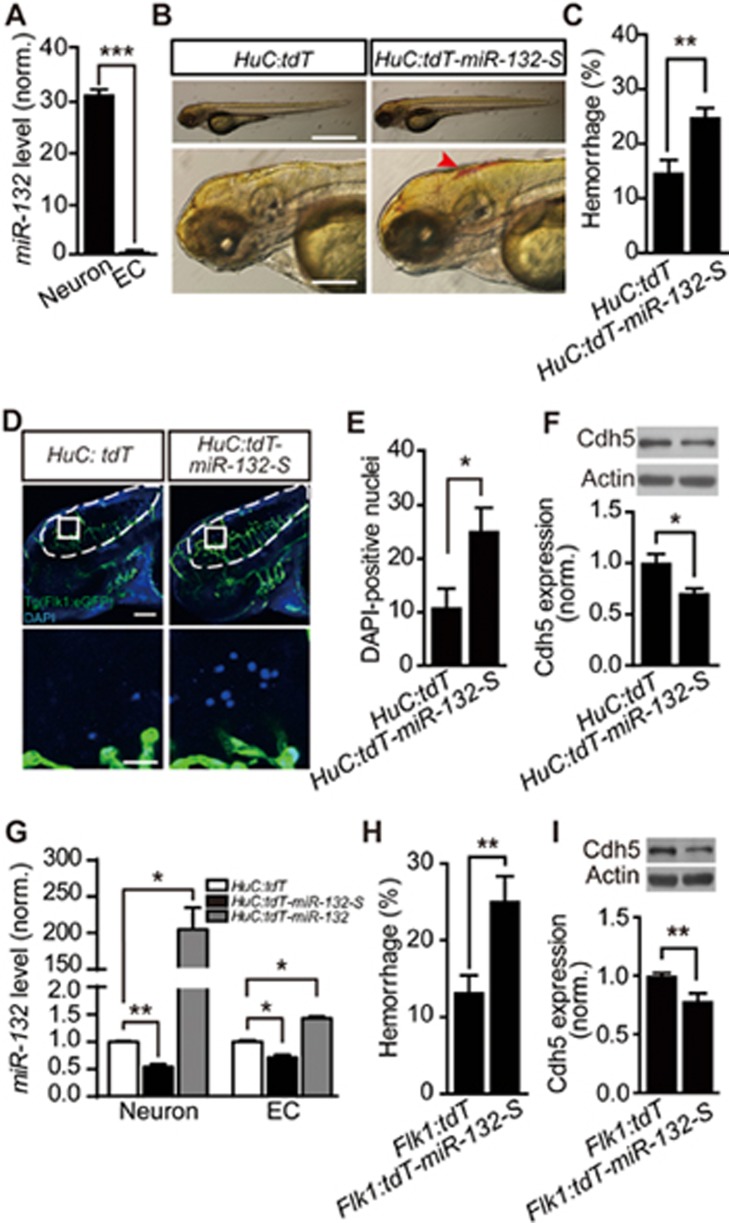
Neuronal *miR-132* regulates brain vascular integrity by affecting endothelial *miR-132* level. **(A)** Relative expression level of *miR-132* in zebrafish neurons and ECs, which were sorted by flow cytometry from 3-dpf Tg(HuC:GFP) or Tg(Flk1:eGFP) larvae, respectively. The experiments were repeated three times. **(B-F)** Effects of *miR-132* sponge (*miR-132-S*)-mediated neuron-specific *miR-132* downregulation on intracranial hemorrhage **(B**, **C)**, DAPI leakage **(D**, **E)**, and Cdh5 expression **(F)** in zebrafish larvae. The sponge was inserted into the *tdTomato* (*tdT*) 3′ UTR and driven by the *HuC* promoter (“*HuC:tdT-miR-132-S*”). Expression of *tdT* in neurons (“*HuC:tdT*”) served as a control. The experiments were repeated 10 times in **C** and 5 times in **F**, and 11 embryos injected with *HuC:tdT* and 8 embryos injected with *HuC:tdT-miR-132-S* were analyzed in **E**. **(G)** Coordinated changes of *miR-132* level in zebrafish neurons and ECs, both of which were sorted from 3-dpf Tg(Flk1:eGFP) larvae transiently expressing *HuC:tdT*, *HuC:tdT-miR-132-S* or *HuC:tdT-miR-132*. Expression of *HuC:tdT-miR-132-S* or *HuC:tdT-miR-132* was used to down- or upregulate *miR-132* level in zebrafish neurons, respectively. The experiments were repeated 3-4 times. **(H**,**I)** Effects of *miR-132-S* expression in ECs on intracranial hemorrhage **(H)** and Cdh5 expression **(I)** in zebrafish larvae. The experiments were repeated nine times in **H** and six times in **I**. For each time, > 14 embryos were examined for each group in **H**. Scale bar, 400 μm (top) and 100 μm (bottom) **(B)**, 100 μm (top) and 20 μm (bottom) **(D)**. Error bars, SEM. ^*^P < 0.05, ^**^P < 0.01, ^***^P < 0.001 (unpaired two-tailed Student's *t*-test for **A**, **C**, **E**, **F**, **H** and **I**; one-way ANOVA with *post hoc* Bonferroni's multiple comparison test for **G**).

In mammals, *miR-132* is enriched in neurons^19,20^. Combining flow cytometry and real-time PCR analysis, we found that, in zebrafish larvae, expression of *miR-132* was much higher in neurons than in ECs (Figure 3A; *P* < 0.001). To study the consequences of downregulating the level of *miR-132* in neurons, we transiently expressed *miR-132* sponge (*miR-132-S*) in neurons. The *miR-132-S* was inserted into the 3′ untranslated region (3′ UTR) of the reporter gene *tdTomato* (*tdT*) and driven by the promoter of the neuron-specific gene *HuC* (“*HuC:tdT-miR-132-S*” Materials and Methods). The sponge contained ten repeats of the *miR-132* antisense sequence and efficiently inhibited *miR-132* function by chelating *miR-132* (Supplementary information, Figure S6; *P* < 0.001)^31^. In comparison with control larvae, in which neurons expressed *tdT* (“*HuC:tdT*”), larvae with neurons expressing *miR-132-S* exhibited severe intracranial hemorrhage (Figure 3B and 3C; *P* < 0.01) and increased DAPI leakage (Figure 3D and 3E; *P* < 0.05) and reduced Cdh5 expression (Figure 3F; *P* < 0.05). Thus, neuronal *miR-132* is necessary for the integrity of brain vasculature. Interestingly, reduction of *miR-132* in neurons by transiently expressing *HuC:tdT-miR-132-S* also decreased the level of *miR-132* in ECs (Figure 3G; *P* < 0.05). Conversely, overexpression of *miR-132* in neurons, through the transient expression of *HuC:tdT-miR-132*, increased the *miR-132* level in ECs (Figure 3G; *P* < 0.05). The non-overlapping expression pattern of *HuC:tdT* and *Flk1:eGFP* ensured that the coordinated changes in endothelial *miR-132* were not due to the non-specific expression of *HuC* plasmids in ECs (Supplementary information, Figure S7). Considering that less than 5% neurons in the brain were labeled by *HuC* promoter-driven transient gene expression, the effects of neuronal manipulations on *miR-132* level in ECs are underestimated.

As ECs are the main constituent of the brain vasculature, we thus proposed that *miR-132* in neurons may regulate the brain vascular integrity by affecting *miR-132* levels in ECs. We then examined whether *miR-132* in ECs can also regulate the brain vascular integrity. To address this point, we specifically downregulated the level of *miR-132* in ECs by transiently expressing *miR-132-S* driven by the promoter of the EC-specific gene *Flk1* (“*Flk1:tdT-miR-132-S*”). In comparison with *tdT* expression in ECs (“*Flk1:tdT*”), *miR-132*-S expression in ECs resulted in severe intracranial hemorrhage (Figure 3H; *P* < 0.01) and reduction of Cdh5 expression (Figure 3I; *P* < 0.01). This indicates that *miR-132* in ECs can regulate expression of Cdh5 and the integrity of the brain vasculature. Together, these results suggest that neuronal *miR-132* may regulate brain vascular integrity through coordinately affecting *miR-132* level in ECs.

### Exosomes transfer neuronal *miR-132* from neurons to ECs

**Figure 4:**
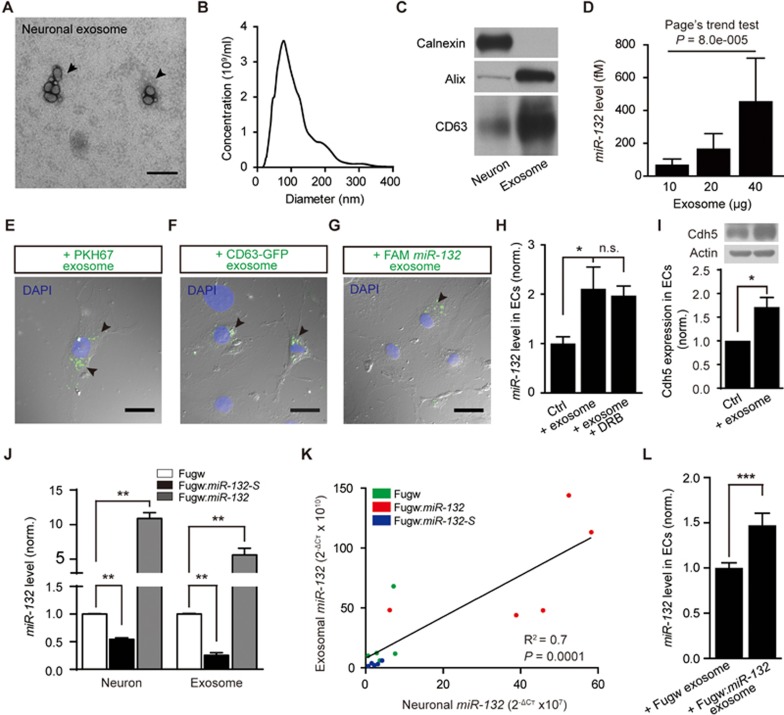
Neuronal exosomes transfer *miR-132* from neurons to ECs. **(A-C)** Characterization of neuron-derived exosomes by transmission electron microscopy (TEM) **(A)**, nanoparticle tracing analysis **(B)** and Western blotting with equal amounts of samples **(C)**. The exosomes were isolated from the conditioned medium of cultured primary rat cortical neurons. Both Alix and CD63 are exosomal markers, and Calnexin is an endoplasmic reticulum marker. **(D)** *MiR-132* level measured with different amount of neuronal exosomes. The experiments were repeated five times. **(E**, **F)** Internalization of PKH67 **(E)** or CD63-GFP **(F)** labeled neuronal exosomes (arrowheads) into the mouse brain microvascular EC line (b.End3). DAPI staining (in blue) was performed to visualize the nuclei of b.End3 cells. **(G)** Existence of FAM-tagged *miR-132* (arrowheads) in b.End3 cells, which were incubated with exosomes isolated from neurons transfected with FAM-tagged *miR-132*. **(H)** Relative level of *miR-132* in b.End3 cells incubated with PBS (“Ctrl”), neuronal exosomes alone (“+ exosome”) (200×), or neuronal exosomes plus the RNA polymerase II inhibitor DRB (“+ exosome + DRB”) (200×). The experiments were repeated nine times. **(I)** Relative expression of Cdh5 in human umbilical vein endothelial cells (HUVECs) incubated with PBS (“Ctrl”) and neuronal exosomes (“+ exosome”) (200×). The experiments were repeated four times. **(J**, **K)** Coordinated changes of *miR-132* level in the cultured rat cortical neurons and their secreted exosomes. The line in **K** represents linear regression with *P* = 0.0001. Cultured neurons were transfected with Fugw:*miR-132-S* or Fugw:*miR-132* to, respectively, down- or up-regulate *miR-132* levels, and transfection with Fugw served as a control. The experiments were repeated five times. **(L)** Relative level of *miR-132* in b.End3 cells incubated with exosomes isolated from neurons transfected with Fugw vector (“Fugw exosome”) or Fugw:*miR-132* (“Fugw:*miR-132* exosome”) (50×). The experiments were repeated three times. Scale bar, 100 nm **(A)**, 25 μm **(E-G)**. Error bars, SEM. n.s., no significant; ^*^P < 0.05, ^**^P < 0.01, ^***^P < 0.001 (Page's trend tests were applied for **D**; one-way ANOVA with *post hoc* Tukey's multiple comparison test for **H** and **J**; unpaired two-tailed Student's *t*-test for **I** and **L**).

Exosomes are newly identified vehicles for intercellular communication^15^. It was reported that neurons can transfer functional materials to recipient cells via secreting exosomes^11,32^ leading us to postulate that neuronal exosomes may transfer *miR-132* from neurons to ECs. To test this hypothesis, we isolated exosomes from the conditioned medium of cultured primary rat cortical neurons (see Materials and Methods). TEM and nanoparticle tracing analysis of the purified pellets identified small membrane vesicles with an average diameter of 112 ± 60 nm (mean ± SD; Figure 4A and 4B), suggesting the identity of exosomes. Furthermore, in comparison with cell lysates, vesicle preparations showed intensive expression of the exosomal markers Alix and CD63 but not the endoplasmic reticulum marker Calnexin (Figure 4C). Importantly, *miR-132* was detected in the isolated neuronal exosome and its level was increased with the increased amount of exosomes (Figure 4D; *P* < 0.001).

To address whether neuronal exosomes containing *miR-132* can be internalized into ECs, we first labeled purified exosomes with the green fluorescent lipid dye PKH67 and incubated them with the mouse brain microvascular EC line (b.End3). After incubation, green fluorescence-positive puncta were observed in both sparse and confluent cultured b.End3 cells (Figure 4E and Supplementary information, Figure S8A). Exosome internalization was further confirmed by incubating b.End3 cells with exosomes purified from neurons in which CD63-GFP fusion protein was expressed. This lead to the appearance of GFP-expressing exosomes in b.End3 cells (Figure 4F). Next, by transfecting neurons with carboxyfluorescein (FAM)-tagged *miR-132*, we observed fluorescence-positive exogenous *miR-132* in neuronal exosome-treated b.End3 cells (Figure 4G). Furthermore, incubation with neuronal exosomes significantly increased the level of *miR-132* in b.End3 cells (Figure 4H; *P* < 0.05). This increase was not affected by co-treatment with the RNA polymerase II inhibitor DRB (5, 6-dichloro-1-b-D-ribofuranosylbenzimidazole; Figure 4H; *P* = 0.9), suggesting neuronal exosomes can transfer mature *miR-132* to ECs. Using a luciferase assay, we found that neuronal exosomes repressed the luciferase activity of a *miR-132* sensor in ECs, indicating that the transferred neuronal exosomal *miR-132* is functional in ECs (Supplementary information, Figure S8B; *P* < 0.05). Consistently, we also found that the expression of Cdh5 in ECs was increased after treatment with neuronal exosomes (Figure 4I; *P* < 0.05). Collectively, these results indicate that neuronal exosomes can transfer *miR-132* into ECs.

By overexpressing or downregulating *miR-132* in cultured cortical neurons, we found that the *miR-132* level in isolated neuronal exosomes positively correlated with that in neurons (Figure 4J and 4K; *P* < 0.01). Moreover, the level of *miR-132* in b.End3 cells could be further increased after incubation with the exosomes isolated from neurons overexpressing *miR-132* (Figure 4L; *P* < 0.001). These *in vitro* findings show that neurons can regulate the level of *miR-132* in ECs through exosome-mediated intercellular transfer of *miR-132*.

**Figure 5:**
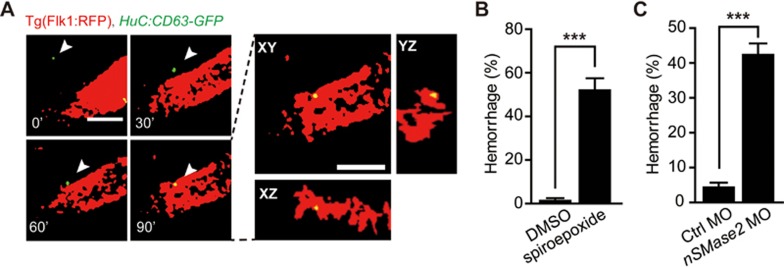
Neuronal exosomes are translocated to brain ECs in larval zebrafish. **(A)** *In vivo* time-lapse confocal images showing that a GFP-positive neuronal exosome (white arrowheads) approaches and enters RFP-positive ECs in the brain. The images were taken from a 3-dpf Tg(Flk1:RFP) zebrafish larva, in which *HuC:CD63-GFP* was transiently expressed to label neuronal exosomes. To trace the moving GFP-positive exosome at each time point, single-slice images at different optical sections are shown at the Left. To confirm the exosome internalization into ECs, three-dimensional views of the single-slice image at 90′ are shown at the Right. **(B**, **C)** Intracranial hemorrhage effects of nSMase2 blockade with spiroepoxide **(B)** or *nSMase2* knockdown with MO **(C)** in zebrafish. The nSMase2 is required for the budding of exosomes into multi-vesicular bodies. The experiments were repeated seven times in **B** and six times in **C**. Scale bar, 5 μm **(A)**. Error bars, SEM. ^***^P < 0.001 (unpaired two-tailed Student's *t*-test for **B** and **C**).

To demonstrate whether neuronal exosomes are involved in the regulation of brain vascular integrity *in vivo*, we labeled neuronal exosomes in zebrafish larvae via transiently expressing CD63-GFP driven by the *HuC* promoter (“*HuC:CD63-GFP*”). The neuron-specific distribution of GFP-positive puncta showed that CD63-GFP can be used to trace neuronal exosomes (Supplementary information, Figure S8C). We then expressed *HuC:CD63-GFP* in Tg(Flk1:RFP) zebrafish larvae, in which vascular ECs expressed red fluorescent protein (RFP). *In vivo* time-lapse confocal imaging showed that GFP-positive neuronal exosomes approached brain vessels and ultimately entered ECs (Figure 5A). To further examine the role of exosomes in regulating brain vascular integrity, we reduced exosome release by targeting neutral sphingomyelinase 2 (nSMase2), which is required for the budding of exosomes into multi-vesicular bodies^33^. Treatment with the nSMase2 inhibitor spiroepoxide (10 μM) or *nSMase2* knockdown with MO led to severe intracranial hemorrhage accompanied with decreased expression of *miR-132* in ECs of zebrafish larvae (Figure 5B, 5C and Supplementary information, Figure S8D-S8F; *P* < 0.05). Taken together, these *in vitro* and *in vivo* findings suggest that neuronal exosomes containing *miR-132* can mediate neuronal regulation of brain vascular integrity.

### *MiR-132* targets *eef2k* in ECs to regulate Cdh5 expression and brain vascular integrity

**Figure 6:**
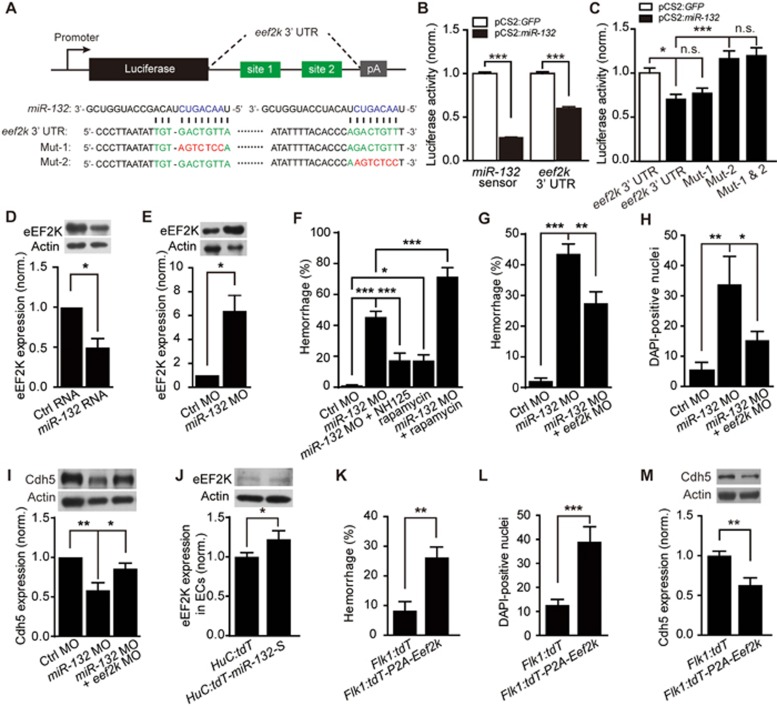
*MiR-132* regulates Cdh5 expression and brain vascular integrity by directly targeting endothelial *eef2k*. **(A)** Schematic showing two potential *miR-132* binding sites (in green) in the *eef2k*3′ UTR. Two mutants (“Mut-1”, “Mut-2”) of the *eef2k*3′ UTR were introduced by replacing the wildtype binding sequence (GACTGTT) with a mutant sequence (AGTCTCC, in red) at the two corresponding binding sites. The seed sequence of *miR-132* is in blue. **(B)** Suppression of the luciferase activity of the *eef2k*3′ UTR reporter in HEK293 cells by *miR-132*. *MiR-132* sensor served as a positive control. The experiments were repeated three times. **(C)** Requirement of the binding site 2 in the *eef2k*3′ UTR for *miR-132* effect. The experiments were repeated three times. **(D**, **E)** Effects of *miR-132* overexpression **(D)** or knockdown **(E)** on eEF2K expression in zebrafish larvae. The experiments were repeated three times. **(F)** Pharmacological evidence showing eEF2K mediates the intracranial hemorrhage effect of *miR-132* knockdown. NH125, eEF2K inhibitor; rapamycin, eEF2K non-specific activator. The experiments were repeated 5-8 times, and for each time, > 15 embryos were examined for each group. **(G-I)** Rescue effect of *eef2k* knockdown on *miR-132* knockdown-induced intracranial hemorrhage **(G)**, DAPI leakage in the brain **(H)** and Cdh5 expression reduction **(I)** in zebrafish larvae. For **G**, the experiments were repeated 6-7 times, and for each time, > 53 embryos were examined for each group. For **H**, 8, 8, and 11 embryos were analyzed for “Ctrl MO”, “*miR-132* MO” and “*miR-132* MO + *eef2k* MO”, respectively. For **I**, the experiments were repeated four times. **(J)** Effect of neuron-specific *miR-132* downregulation on endothelial eEF2K expression in zebrafish larvae. ECs were sorted by flow cytometry from 3-dpf Tg(Flk1:eGFP) larvae, which were injected with *HuC:tdT* or *HuC:tdT-miR-132-S* plasmid. The experiments were repeated three times. **(K-M)** Effects of EC-specific *Eef2k* overexpression on intracranial hemorrhage **(K)**, DAPI leakage in the brain **(L)**, and Cdh5 expression **(M)** of zebrafish larvae. The *Eef2k* was cloned into *Flk1:tdT-P2A* vector (“*Flk1:tdT-P2A-Eef2k*”). Expression of *tdT* in ECs (“*Flk1:tdT*”) served as a control. For **K**, the experiments were repeated nine times, and for each time, > 5 embryos were examined for each group. For **L**, 12 and 13 embryos were analyzed for “*Flk1:tdT*” and “*Flk1:tdT-P2A-Eef2k*”, respectively. For **M**, the experiments were repeated six times. Error bars, SEM. n.s., no significant; ^*^P < 0.05, ^**^P < 0.01, ^***^P < 0.001 (unpaired two-tailed Student's *t*-test for **B**, **D**, **E**, **J-M**; one-way ANOVA with *post hoc* Tukey's multiple comparison test for **C** and **I**; one-way ANOVA with *post hoc* Bonferroni's multiple comparison test for **F**, **G** and **H**).

To search for the direct target of *miR-132* that is involved in the regulation of brain vascular integrity, we identified 1 253 upregulated genes in *miR-132* morphants by performing microarray analysis (accession number for the microarray data: GSE85291 in NCBI) and predicted 848 target genes by using the Microcosm database. Among these predicted targets, 21 candidate genes (12 annotated) overlapped (Supplementary Information, Figure S9A). We then performed an *in vitro* luciferase assay for the 12 annotated genes. Consistent with the microarray data and the prediction that the zebrafish *eef2k* 3′UTR contains two *miR-132* binding sites (Figure 6A), we found that *miR-132* markedly repressed the luciferase activity of the *eef2k* 3′UTR (Supplementary Information, Figure S9B). The *eef2k* encodes a calcium/calmodulin-dependent protein kinase, which phosphorylates eukaryotic elongation factor 2 (eEF2) to inhibit translational elongation^34,35^. Both *in vitro* and *in vivo* reporter assays showed that *eef2k* 3′ UTR was sufficient to confer *miR-132* regulation (Figure 6B and Supplementary information, Figure S10A and S10B; *P* < 0.001), and the second binding site was indispensable (Figure 6A and 6C). Importantly, the expression of eEF2K in zebrafish larvae was decreased or increased by the overexpression or knockdown of *miR-132*, respectively (Figure 6D and 6E; *P* < 0.05). These results indicate that *eef2k* is a direct target of *miR-132*.

We then examined whether eEF2K mediates the effect of *miR-132* on the brain vascular integrity. Application of the eEF2K inhibitor NH125 (2.5 μM) significantly alleviated the hemorrhagic defect in *miR-132* morphants (Figure 6F; *P* < 0.001). On the other hand, treatment with the eEF2K non-specific activator rapamycin (2 μM) led to intracranial hemorrhage in wildtype larvae (Figure 6F; *P* < 0.05) and further aggravated the hemorrhagic defect of *miR-132* morphants (Figure 6F; *P* < 0.001). Moreover, knockdown of *eef2k* alleviated the intracranial hemorrhage (Figure 6G; *P* < 0.01), DAPI leakage (Figure 6H and Supplementary information, Figure S10C; *P* < 0.05) and reduction of Cdh5 expression (Figure 6I; *P* < 0.05) in *miR-132* morphants. More importantly, by combining flow cytometry and Western blotting, we found that the expression of eEF2K in ECs was significantly increased after sponge-mediated reduction of *miR-132* in neurons (Figure 6J; *P* < 0.05). Furthermore, EC-specific overexpression of *Eef2k* (“*Flk1:tdT-P2A-Eef2k*”) also caused intracranial hemorrhage (Figure 6K; *P* < 0.01), DAPI leakage (Figure 6L and Supplementary information, Figure S10D; *P* < 0.001), and reduction of Cdh5 expression (Figure 6M; *P* < 0.01). Taken together, these results indicate that *eef2k* is a direct target of *miR-132* in ECs to regulate the integrity of brain vasculature.

## Discussion

**Figure 7:**
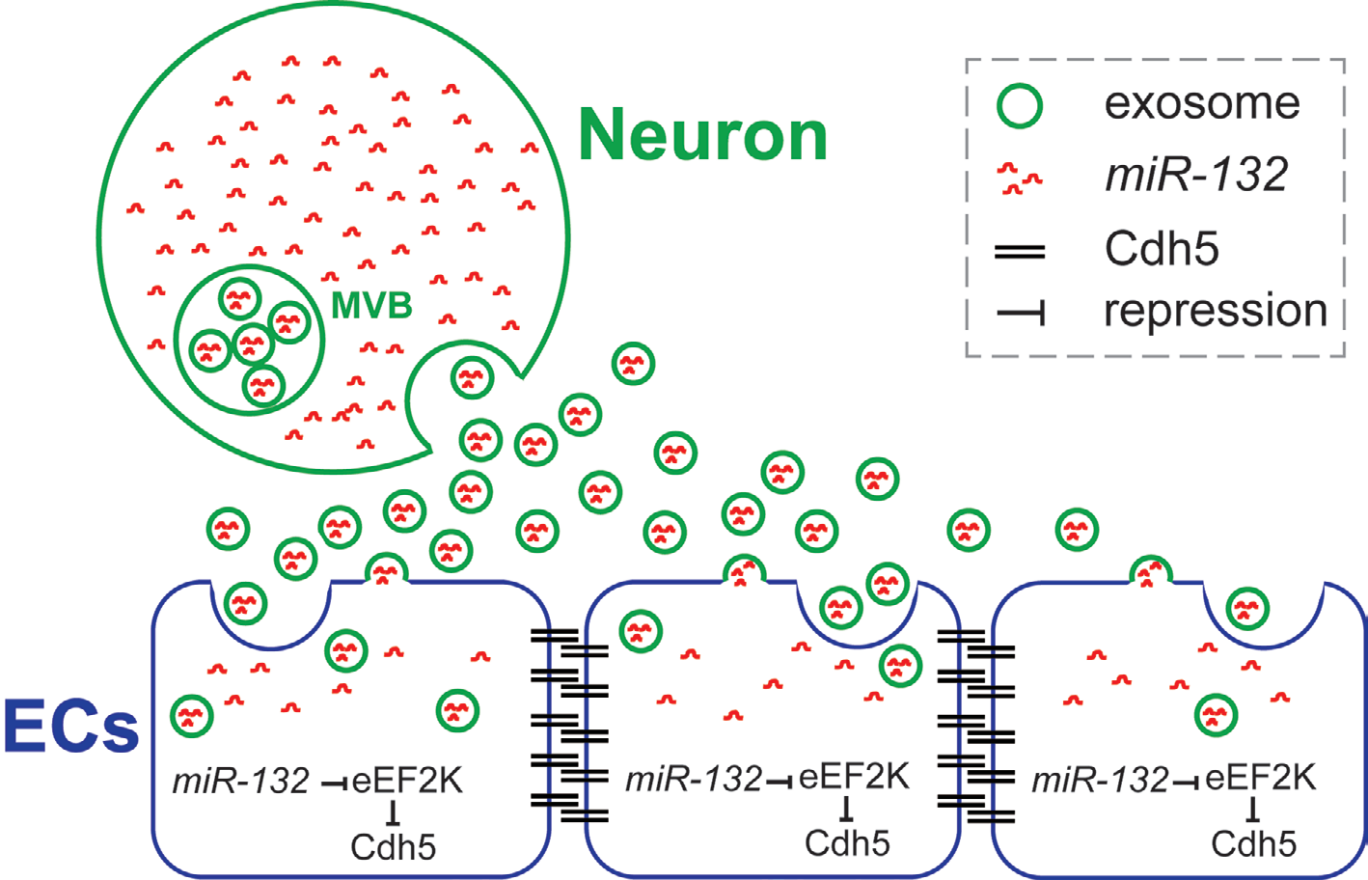
Working model. Neurons secrete *miR-132*-containing exosomes, which are internalized into brain ECs. In ECs, *miR-132* upregulates Cdh5 expression via directly repressing eEF2K expression, promoting the development of brain vascular integrity.

Our study identifies a novel mechanism for the neural regulation of brain vascular integrity. Through secreting exosomes, neurons can translocate *miR-132* to ECs to regulate the expression of the vascular junction protein Cdh5 by targeting *eef2k* (Figure 7). It highlights that neuronal exosomes can serve as an important mediator for neurovascular communication.

### Novel function of *miR-132* in regulating brain vascular integrity

*MiR-132*, a neuron-enriched miRNA, plays important roles in the development and activity-dependent plasticity of neural systems in a cell-autonomous manner. At a cellular level, *miR-132* regulates neurite growth and arborization^20,36,37^, and synaptic structure and function^19,38^. At a system level, *miR-132* is essential for experience-dependent structural and functional plasticity of the visual cortex^39,40^. In the present study, we found that *miR-132* exerts a non-cell-autonomous function in regulating the integrity of the brain vasculature. It can be transferred from neurons to brain ECs to promote the brain vascular integrity. As the impairment of brain vascular integrity will lead to the ingress of plasma components into the brain and compromise synaptic and neuronal functions^1,2^, the regulation of brain vascular integrity by *miR-132* may indirectly contribute to the role of *miR-132* in neural development and function.

The brain microenvironment is so complicated that different cell types and molecules are coordinated to regulate the development of brain vasculature. Besides neurons, pericytes and astrocytes are important for the development and maintenance of the integrity of brain vasculature^29,30,41^. However, there is no direct evidence for the existence of astrocytes in zebrafish larvae^42^. Here we found that knockdown of *miR-132* did not affect the pericyte coverage, suggesting no direct involvement of pericytes in the regulation of brain vascular integrity by *miR-132*. However, previous reports showed that *miR-132* was also expressed in pericytes and glial cells^43,44^. Thus, it is of interest to examine whether *miR-132* in pericytes and glial cells can also regulate brain vascular integrity.

### Emerging roles of neural systems in brain vascular integrity

During development, a few neural tissue-derived proteins were found to promote brain vascular development^4,5^. During embryogenesis, neural progenitor cells can secrete Wnt ligands to regulate the development of brain vasculature and BBB through activation of canonical Wnt signaling in ECs^6,7^. During postnatal development, astrocytes can release Shh to promote the brain vascular integrity by acting on EC Patched-1 receptors^9^. In addition, both pericytes and astrocytes can release Angiopoietin-1 that binds to endothelial Tie-2 receptors to improve brain vascular integrity^45,46^. Our study uncovers a new type of signal, i.e. neuron-derived miRNA, which is involved in the neural regulation of brain vascular integrity. As the expression of *miR-132* is positively regulated by sensory experience and neural activity^38,39,40^, it is of interest to examine whether enriched environment or enhanced neural activity can promote the development of brain vascular integrity by increasing *miR-132* levels. As *miR-132* expression is kept at a high level in the brain^39^, it is possible that the mechanism we have identified may also contribute to the maintenance of brain vascular integrity in adult brain.

### Exosomes are a novel avenue for neurovascular communication

Increasing evidence indicates that exosome-mediated intercellular communication plays important roles in both physiological and pathological processes in neural systems^16,17^. Under physiological conditions, neurons can transfer *miR-124a* in exosomes to astrocytes to regulate the expression of astroglial glutamate transporters, which is important for the maintenance of extracellular glutamate levels^47^. Synapsin 1-containing exosomes secreted by astrocytes can be taken up by neurons and promote neurite outgrowth and neuronal survival^48^. Exosomes released by Schwann cells and oligodendrocytes can be taken up by neuronal axons to increase neurite growth, enhance axonal regeneration and improve neuronal survival^49,50^. Under pathological conditions, exosomes have been found to increase the propagation of neurotoxic proteins in neurodegenerative diseases^51,52,53^, and aggravate brain tumor metastasis and progression^14^. During neural development, cultured embryonic rat cortical neurons can release exosomes^54^. *In vivo* studies in *Drosophila* larvae showed that motor neurons can secrete Wingless-associated and Synaptotagmin 4-containing exosomes to promote the synaptic development and plasticity^11,18^. In the present study, we showed that neuronal exosomes can also be internalized by brain ECs to mediate the neural regulation of brain vascular development. As release of neuronal exosomes can be upregulated by increased neural activity^32,54^, neural activity may regulate the brain vascular development and permeability through exosome release. As the contents of exosomes are abundant, other factors besides *miR-132* in neuronal exosomes may also participate in the neural regulation of brain vascular integrity. It will be of great future interest to examine the roles of other agents present in neuronal exosomes in neurovascular crosstalk.

The exosome is a suitable vehicle for drug delivery because of its high biocompatibility and low immunogenicity^55^. In addition, formulated nanoparticle-mediated *miR-132* delivery into ECs can improve EC survival *in vitro* and EC transplantation efficiency in mice^56^. Therefore, application of *miR-132*-containing exosomes may be a safe and efficient future strategy for alleviating brain vascular dysfunctions associated with neurological and brain vascular diseases.

Our findings open an exciting avenue for the study of neuronal exosome-mediated neurovascular interactions. Further elucidation of neuronal exosomal functions in the regulation of brain vasculature should be of important biological and clinical significance.

## Materials and Methods

### Zebrafish husbandry

Adult zebrafish (*Dario rerio*) were maintained with an automatic fish housing system (ESEN, China) at 28 °C following standard protocols. The AB/WT, Tg(Flk1:eGFP), Tg(Flk1:RFP), Tg(Gata1:DsRed) and Tg(HuC:GFP) zebrafish were described previously^57,58^. Zebrafish embryos were raised under a 14 h-10 h light-dark cycle in 10% Hanks' solution that consisted of 140 mM NaCl, 5.4 mM KCl, 0.25 mM Na_2_HPO_4_, 0.44 mM KH_2_PO_4_, 1.3 mM CaCl_2_, 1.0 mM MgSO_4_, and 4.2 mM NaHCO_3_ (pH = 7.2). 0.003% 1-phenyl-2-thiourea (PTU) (Sigma, P7629) was added in the Hanks' solution to prevent pigment formation of zebrafish embryos. Zebrafish chorion was removed at 1 dpf with the treatment of 2 mg/ml pronase (Calbiochem, 53702), which was diluted in the Hanks' solution. All animal use and handling procedures were approved by Institute of Neuroscience, Chinese Academy of Sciences.

### Morpholino oligonucleotides and microinjection

Morpholino oligonucleotides (MOs) were purchased from Gene Tools (Philomath, OR). Lyophilized MOs were dissolved in nuclease-free water. Zebrafish embryos were microinjected at the one-cell stage with 8 ng *miR-132* MO, 8 ng *miR-132* loop MO, 0.5 ng *Cdh5* MO, 0.5 - 1 ng *eef2k* MO, 4 ng *nSMase2* MO, or equivalent control MO. In *miR-132* rescue experiments, 8 ng *miR-132* MO was co-injected with 20 nM *miR-132* RNA or control RNA (Genepharma). The sequences of MOs or RNAs used are as follows.

*miR-132* MO: 5′-AGCGACCATGGCTGTAGACTGTTAC-3′

*miR-132* loop MO: 5′-GGCTGTAGACTGTTACCAAAAATTC-3′

*Cdh5* MO: 5′-TACAAGACCGTCTACCTTTCCAATC-3′

*nSMase2* MO: 5′-CCACCTGCACCTGCACAAAACAACA-3′

*eef2k* MO: 5′-AGCTCCTCTTCAGCCATGATGCCCC-3′

control MO: 5′-CCTCTTACCTCAGTTACAATTTATA-3′

*miR-132* RNA: 5′-UAACAGUCUACAGCCAUGGUCG-3′

control RNA: 5′-UUGUACUACACAAAAGUACUG-3′

### CRISPR/Cas9-mediated mutation of *miR-132* in zebrafish embryos

The CRISPR (clustered regularly interspaced short palindromic repeats)/Cas9 system was applied to introduce *miR-132* gene mutation in zebrafish embryos as previously reported^58,59,60^. The zebrafish codon-optimized *Cas9* (z*Cas9*) expression plasmid pGH-T7-z*Cas9* was linearized by *XbaI* and used as a template for z*Cas9* mRNA synthesis *in vitro* with mMACHINE T7 Ultra kit (Ambion, AM1345). The sequence of *miR-132* guide RNA (gRNA) (5′-TTGGTAACAGTCTACAGCCA-3′) was designed to target the sequence of mature *miR-132* (Supplementary information, Figure S4A). A pair of oligonucleotides containing the *miR-132* gRNA sequence were annealed and cloned into the pT7-gRNA plasmid. Then *miR-132* gRNA was synthesized with MAXIscript T7 kit (Ambion, AM1312M) and purified with mirVana miRNA isolation kit (Ambion, AM1560). 600 pg z*Cas9* mRNA and 100 pg *miR-132* gRNA were co-injected into zebrafish embryos at one-cell stage. The *miR-132* gene mutation in F0 embryos was examined by PCR and sequencing analysis with primers (forward: 5′-CGCCTCGAGCAGTCTACAGTCATGGCTACTGACG-3′ reverse: 5′-GCGTCTAGACCTGTTCACTTGCATGCAAGG-3′). The knockout efficiency of mature *miR-132* transcription was examined by real-time PCR, and hemorrhagic phenotypes were examined in F0 embryos which carried *miR-132* mutations.

### Generation of pericyte reporter zebrafish line Ki(pdgfrβ:Gal4)

The Ki(pdgfrβ:Gal4) line was generated with CRISPR/Cas9-mediated knockin method as previously reported^58^. Briefly, the *pdgfrβ* gRNA, which targets to the intron between the last and second last exons, was synthesized. For the donor plasmid, the left and right arms of *pdgfrβ* were amplified from the zebrafish genomic DNA. The left arm, gal4 fragment and right arm were ligated into the PM19-T vector. 1 nl RNAase free liquid mixture, which contained 800 pg *zCas9* mRNA, 80–100 pg *pdgfrβ* gRNA and 15 pg *pdgfrβ* donor plasmid, was injected into zebrafish embryos at one-cell stage. The embryos were raised to adulthood for founder screening. To screen Ki(pdgfrβ:Gal4) founders, adult fish were crossed with AB/WT zebrafish, the genomic DNA was extracted at 1 dpf, and the germline transition was detected by PCR.

### *In vivo* confocal imaging

Larvae at 3-dpf were embedded in 1% low-melting agarose (Invitrogen, 16520-050) without anesthetic at room temperature. Imaging was carried out with an Olympus Fluoview 1000 confocal microscopy (Olympus, Japan). Lumplfl 40× (W/IR; NA, 0.80) and XLumplfl 20× (W/IR; NA, 0.95) objective lenses (Olympus) were used. The z-step of imaging ranged from 2 to 3 μm. To detect the internalization of CD63-GFP-labeled neuronal exosomes into brain endothelial cells (ECs) in intact larvae, a Lumplfl 40× (W/IR; NA, 0.80) objective lens was used with a z-step of 0.5 μm. Time-lapse images were taken with a 15-min interval. Raw images were processed with deconvolution (Huygens Essential) to reduce the convolution effect produced by microscope optics and then thresholding with the Triangle method (ImageJ).

### Detection of brain vascular integrity

10 nl mixture of DAPI (0.8 mg/ml) and 10-kDa Alex-568 dextran (10 mg/ml) was injected into the blood circulation system of 3-dpf zebrafish larvae through the common cardinal vein. After 30 min, images were taken for calculating the number of DAPI-positive parenchymal nuclei outside brain vessels as previously described^24^. To detect leakage sites, 100-nm red fluorescent beads were injected into the blood circulation system and time-lapse images were taken immediately after the injection.

### Transmission electron microscopy

Larvae at 3 dpf were fixed in 2% glutaraldehyde in 0.1 M phosphate buffer (pH = 7.4) overnight at 4 °C. Preparations were then washed, post-fixed with 1% osmium tetraoxide, dehydrated and embedded. Sections (70 nm in thickness) were cut and observed by transmission electron microscopy (JEM-1230).

### Calculation of pericyte coverage of brain blood vessels

The numbers of brain blood vessel segments and pericytes in the zebrafish brain were quantitatively analyzed by using Brain Angiotome 1.1^57^. The pericyte coverage of brain blood vessels was defined as the average number of pericytes for each brain blood vessel segment.

### Flow cytometry

To compare the expression level of *miR-132* in zebrafish ECs and neurons, Tg(Flk1:eGFP) and Tg(HuC:GFP) larvae were used for EC and neuron sorting, respectively. To examine the effect of neuronal *miR-132* manipulations on the expression of *miR-132* and eEF2K in ECs, Tg(Flk1:eGFP) larvae microinjected with *HuC:tdT, HuC:tdT-miR-132 or HuC:tdT-miR-132-S* were used for both neuron and EC sorting. Zebrafish embryos were dechorionated with pronase at 1 dpf. Embryos at 3 dpf were suspended with PBS containing 2% Fetal Bovine Serum (FBS), and pipetted up and down gently to remove the yolk. Embryos were then digested with 0.25% trypsin at 28 °C for 30 min. Dissociated cells were collected by centrifugation, suspended with PBS containing 2% FBS, and then filtered with 40-μm cell strainer (BD Falcon, 352340). The cell suspension was used to sort out neurons or ECs by a MoFlo XDP flow cytometer (Beckman, ML99030) with 488-nm and 560-nm lasers.

### Rat primary cortical neuron culture and transfection

Primary cortical neurons were prepared from E16 Sprague-Dawley rat embryos as previously reported^54^. In brief, cerebral cortices were dissected and digested with 0.125% trypsin at 37 °C for 10 min. Dissociated cells were filtered with 70-μm cell strainer (BD Falcon, 352360), collected by centrifugation and then plated onto Petri dishes coated with 0.01 mg/ml Poly-D-Lysine (Sigma, P6407). Neurons were maintained at 37 °C in a humidified atmosphere of 5% CO_2_ and cultured in Neurobasal medium (Invitrogen, 21103049) supplemented with Glutamax (Invitrogen, 35050061), B27 (Invitrogen, 17504044) and 5% FBS (Biochrom, S0115/S0615) for the first day. From the next day, neurons were cultured in serum-free medium and half of the medium was replaced every 3 days. To manipulate the expression of *miR-132* or label exosomes in cultured neurons, dissociated rat cortical neurons were transfected with 2 μg plasmid or 100 nM FAM-tagged *miR-132* (Genepharma) with a Rat Neuron Nucleofector Kit (Amaxa, VPG1003) by using an electroporator (Amaxa, Program O-03). The transfected cells were plated onto Poly-D-Lysine coated 6-well plates and cultured as described above.

### Purification, identification, labeling and treatment of rat neuronal exosomes

Neuronal exosomes were purified from the conditioned medium of rat cortical neurons cultured 5 or 9 days *in vitro* (DIV). All transfected neurons were cultured 5 DIV in culture plates and exosomes were isolated by Total Exosome Isolation Reagent (Invitrogen, 4478359). Untransfected neurons were cultured 9 DIV in petri dishes and exosomes were isolated by multi-step centrifugation as previously described^61^. The isolated pellets were suspended in 1×PBS for exosome characterization and culture experiments. For electron microscopy, 10 μl suspended pellets were applied onto carbon-coated copper grids. After the sample was dry, micrographs were taken with a calibrated magnification of 100 000 fold by transmission electron microscope (JEM-1230). For nanoparticle tracking analysis, suspended pellets were diluted in 1 ml PBS and applied to NanoSight (NS 300) to automatically measure the average diameter and concentration. To examine the uptake of neuronal exosomes by ECs *in vitro*, neuronal exosomes were labeled using a PKH67 green fluorescent labeling kit (Sigma-Aldrich, MINI67) or isolated from rat cortical neurons transfected with Fugw:*CD63-GFP* plasmid. The labeled neuronal exosomes were then incubated with the mouse brain microvascular EC line (b.End3) at 37 °C for 12 h. The exosome-treated b.End3 cells were then fixed and imaged. To examine the level of endothelial *miR-132* after neuronal exosome treatment, b.End3 cells were incubated with the isolated neuronal exosomes at 37 °C for 24 h. The addition of exosomes isolated from 50 ml neuronal conditioned media into 0.5 ml ECs culture medium was counted as concentrating 100 times (100×).

### Plasmid constructions

To increase the expression of *miR-132*, zebrafish pri-*miR-132* was amplified from the zebrafish genomic DNA by using primers (forward: 5′-CGCCTCGAGCAGTCTACAGTCATGGCTACTGACG-3′ reverse: 5′-GCGTCTAGACCTGTTCACTTGCATGCAAGG-3′) and cloned into *HuC:tdT*, pCS2:*GFP* or Fugw vectors. To reduce *miR-132* expression, *miR-132* sponge (*miR-132-S*) containing 10 repeated *miR-132* antisense sequences was synthesized by GeneArt and cloned into *HuC:tdT*, *Flk1:tdT* or Fugw vectors. The *Eef2k* was amplified from mouse cDNA with primers (forward: 5′-CGCCTTAAGCAAGCTGGTGATGTTGAAGAAAATCCTGGTCCTATGGCAGACGAAGACCTCATCTTC-3′ reverse: 5′- CGCGGATCCCGCTCTAGATTATTCCTCCATCTGGGCCCA-3′) and cloned into *Flk1:tdT-P2A* vector. To label neuronal exosomes *in vivo* and *in vitro*, human *CD63-GFP* (System Biosciences, CYTO120-VA-1) was cloned into *HuC* or Fugw vectors. All clones were verified by sequencing analysis. 30 ng plasmid was injected into zebrafish embryos at one-cell stage.

### Whole-mount *in situ* hybridization

Zebrafish whole-mount *in situ* hybridization was performed as previously described^22,62^ with a digoxigenin-labeled *miR-132* Locked Nucleic Acid (LNA) probe (5′-CGACCATGGCTGTAGACTGTTA -3′, Exiqon) and a scramble LNA probe (5′-GTGTAACACGTCTATACGCCCA-3′, Exiqon). Embryos were incubated with corresponding probes (1:500) at 49 °C overnight. Digoxigenin was detected by anti-Digoxigenin AP-conjungated antibody (1:5 000, Roche 11093274910) and developed with NBT/BCIP solution (Roche, 11681451001). Images were taken with an upright metallurgical microscope (Zeiss).

### Immunofluorescence

Zebrafish whole-mount immunofluorescence was performed as previously reported^63^. Briefly, 2-dpf embryos were fixed in 4% paraformaldehyde at 4 °C overnight. Then embryos were washed 4 × 5 min in PBST (PBS + 0.5% Triton X-100) and incubated in a blocking solution (PBST + 10% normal goat serum + 1% BSA) for 2 h at room temperature. Embryos were then incubated with a rabbit anti-Cdh5 primary antibody (1:500, from Dr M Affolter's Lab) in the blocking solution at 4 °C overnight. Embryos were further washed 6 × 30 min in PBST and then incubated with an Alexa-568 goat anti-rabbit IgG secondary antibody (1:1000, Invitrogen) in the blocking solution at 4 °C overnight. After washing 3-5 times in PBST, images were taken with an Olympus Fluoview 1000 confocal microscope (Olympus, Japan).

### Western blotting

Zebrafish embryos, cultured rat cortical neurons and isolated exosomes were lysed in RIPA buffer (Beyotime, P0013D) containing Protease Inhibitor Cocktail (Merck, 539134). Protein samples for CD63 detection were extracted by using Triton X-100 lysis buffer (20 mM Tris·HCl at pH 7.4, 137 mM NaCl, 1% Triton X-100, 2 mM EDTA, and 10% glycerol) containing Protease Inhibitor Cocktail^12^. Protein concentration was measured by a Bio-Rad Protein Assay Kit and equal amounts of proteins were separated by SDS-PAGE and transferred onto PVDF membranes. Membranes were incubated in a blocking solution (TBS + 0.2% Triton X-100 + 5% BSA) for 2 h at room temperature and then probed with primary antibodies (as indicated below) overnight at 4 °C. After washing 3 × 15 min in TBST (TBS + 0.2% Triton X-100), membranes were incubated with HRP-conjugated secondary antibodies (Jackson) for 1 h at room temperature, rewashed and signals detected with the ECL Western blotting substrate (Pierce). The primary antibodies were anti-Cdh5 (Cell Signaling, 2500), anti-α-catenin (BD Pharmingen, 610193), anti-β-catenin (Abcam, ab6302), anti-ZO1 (Invitrogen, 40-2200), anti-Claudin-5 (Invitrogen, 187364), anti-Occludin (Invitrogen, 71-1500), anti-N-cadherin (Invitrogen, 33-3900), anti-eEF2K (Cell Signaling, 3692), anti-Actin (Abmart, M20010), anti-CD63 (BD Pharmingen, 551458), anti-Calnexin (Abcam, ab22595), and anti-Alix (Cell Signaling, 2171).

### *In vitro* luciferase and *in vivo* reporter assays

Two repeated *miR-132* antisense sequences were cloned into psiCHECK-2 vector as a *miR-132* sensor. Zebrafish *eef2k* 3′ UTR (500 bp) containing *miR-132* target sequences was amplified from the zebrafish cDNA with primers (forward: 5′-CGCCTCGAGTTTACAGATTGATCAAAATGGTTTA-3′ reverse: 5′-CGCTCTAGACGCGCGGCCGCTCCAGCAAAAGTGATCACACA-3′) and cloned into psiCHECK-2 or psiCHECK-2*:tdTomato* vector. The mutant *eef2k* 3′ UTR with mis-matched *miR-132* binding sites was amplified using mutant primers. For *in vitro* luciferase assay, 100 ng luciferase reporter plasmid was co-transfected with 400 ng pCS2:*GFP* or pCS2:*miR-132* plasmid into cultured HEK293 cells using Lipofectamine 2000 (Invitrogen, 11668027). Luciferase activity was detected with a Dual-Luciferase Reporter Assay System (Promega, E1910) 24 h after transfection. For the *in vivo* reporter assay, linearized psiCHECK-2:*tdTomato-eef2k* 3′ UTR and pCS2:*GFP* plasmids were used as the templates for *tdTomato-eef2k* 3′UTR and *GFP* mRNAs synthesis *in vitro*, respectively. The *tdTomato-eef2k* 3′UTR mRNA (50 pg) and *GFP* mRNA (30 pg), together with control RNA (200 pg) or *miR-132* RNA (200 pg), were co-injected into zebrafish embryos at the one-cell stage. Images were taken at 1 dpf and the ratio of tdTomato to GFP fluorescence intensity was calculated with ImageJ.

### RNA preparation and real-time PCR

Total RNAs of zebrafish embryos, flow cytometry-sorted zebrafish neurons and ECs, cultured cells and isolated exosomes were extracted by using TRIzol reagent according to the manufacturer's instructions (Invitrogen, 15596018). The extracted total RNA was used to generate the first-strand cDNA by using PrimeScript reverse transcriptase (Takara, 2680A) with specific stem-loop primer for *miR-132* and random primer. The real-time PCR with SYBR Premix Ex Taq II (Takara) was performed on the cDNA to detect the relative *miR-132* expression. The relative RNA amount was calculated with the ΔΔCt method and normalized with U6 expression (as an internal control). The Taqman real-time PCR was only used to detect the absolute quantity of *miR-132* in different amount of neuronal exosomes. The primers used for real-time PCR are as follows.

*miR-132* stem-loop primer:

5′-TGGAGCGACCGTGTCGTGGAGTCGGCTAATGGTCGCTCCACGACC-3′

*miR-132* primers:

forward: 5′-GACACTCCAGCAGCGTAACAGTCTACAGCCATG-3′

reverse: 5′-ATAGAGCGGTGTCGTGGAGTCGGCTAATGGTC-3′

zebrafish U6 primers:

forward: 5′-ACTAAAATTGGAACGATACAGAGA-3′

reverse: 5′-AAAGATGGAACGCTTCACG-3′

mouse U6 primers:

forward: 5′-TCGCTTCGGCAGCACATA-3′

reverse: 5′-ACGAATTTGCGTGTCATCCT-3′

### Drug treatment

1-Benzyl-3-cetyl-2-methylimidazolium iodide (NH125) (2.5 μM, Tocris 3439), rapamycin (2 μM, Merck 553211), spiroepoxide (10 μM, Santa Cruz SC-202721) or equivalent DMSO was added into zebrafish culture medium at 1 dpf after the chorion was removed. The medium was replaced with fresh medium containing corresponding drugs at 2 dpf and phenotypes were characterized at 3 dpf.

### Statistical analysis

The significance of difference between two groups was determined by using unpaired two-tailed Student's *t*-test. For multiple-group comparisons, one-way ANOVA was performed along with *post hoc* Bonferroni's or Tukey's multiple comparison test to assess statistical significance with a 95% confidence interval. Bonferroni's Multiple Comparison Test was used for groups with different experimental numbers, while Tukey's Multiple Comparison Test was used for groups with same experimental numbers. Page's trend tests were applied for examining whether there is an increasing trend in the level of *miR-132* as the amount of exosomes increases in Figure 4D. Calculations were performed by using GraphPad Prism v5.0 software. Data were represented as mean ± SEM in all figures except specifically indicated, and *P* < 0.05 was considered to be statistically significant.

### Accession number

The accession number for the microarray data is GEO: GSE85291 (http://www.ncbi.nlm.nih.gov/geo/).

## Author Contributions

JLD and BX conceived the project. BX, YZ and JLD designed the experiments. BX and YZ carried out the experiments and analyzed the data. XFD helped find the preliminary phenomenon. JL, HXZ, YY and JWB contributed to the generation of knockout and knockin zebrafish lines. HH contributed to TEM-relevant experiments. JLD, BX and YZ wrote the manuscript.

## Competing Financial Interests

The authors declare no competing financial interests.

## Supplementary Information

Supplementary information, Figure S1 Conserved *miR-132* is necessary for brain vascular integrity.

Supplementary information, Figure S2 *MiR-132* knockdown reduces brain vascular density.

Supplementary information, Figure S3 Impairment of brain vascular integrity in nonhemorrhagic *miR-132* morphants.

Supplementary information, Figure S4 Mutation of *miR-132* by CRISPR/Cas9.

Supplementary information, Figure S5 *Cdh5* knockdown impairs brain vascular integrity in larval zebrafish.

Supplementary information, Figure S6 Design and efficiency of *miR-132* sponge.

Supplementary information, Figure S7 No overlapping between *HuC*-driven tdTomato expression in neurons and *Flk1*-driven eGFP expression in ECs.

Supplementary information, Figure S8 Internalization of neuronal exosomes into ECs and effect of impaired exosome release on zebrafish intracranial hemorrhage.

Supplementary information, Figure S9 Prediction of the target genes of *miR-132* in zebrafish.

Supplementary information, Figure S10 Role of *eef2k* in mediating *miR-132* effects on brain vascular integrity.
