# Supplementary material for: Neurons secrete miR-132-containing exosomes to regulate brain vascular integrity
Source: Cell Res. 2017 Apr 21;27(7):882–97. doi: 10.1038/cr.2017.62 (PMC5518987; doi:10.1038/cr.2017.62)
Supplement: Supplementary information, Figure S1 — Conserved miR-132 is necessary for brain vascular integrity. [file cr201762x1.pdf]

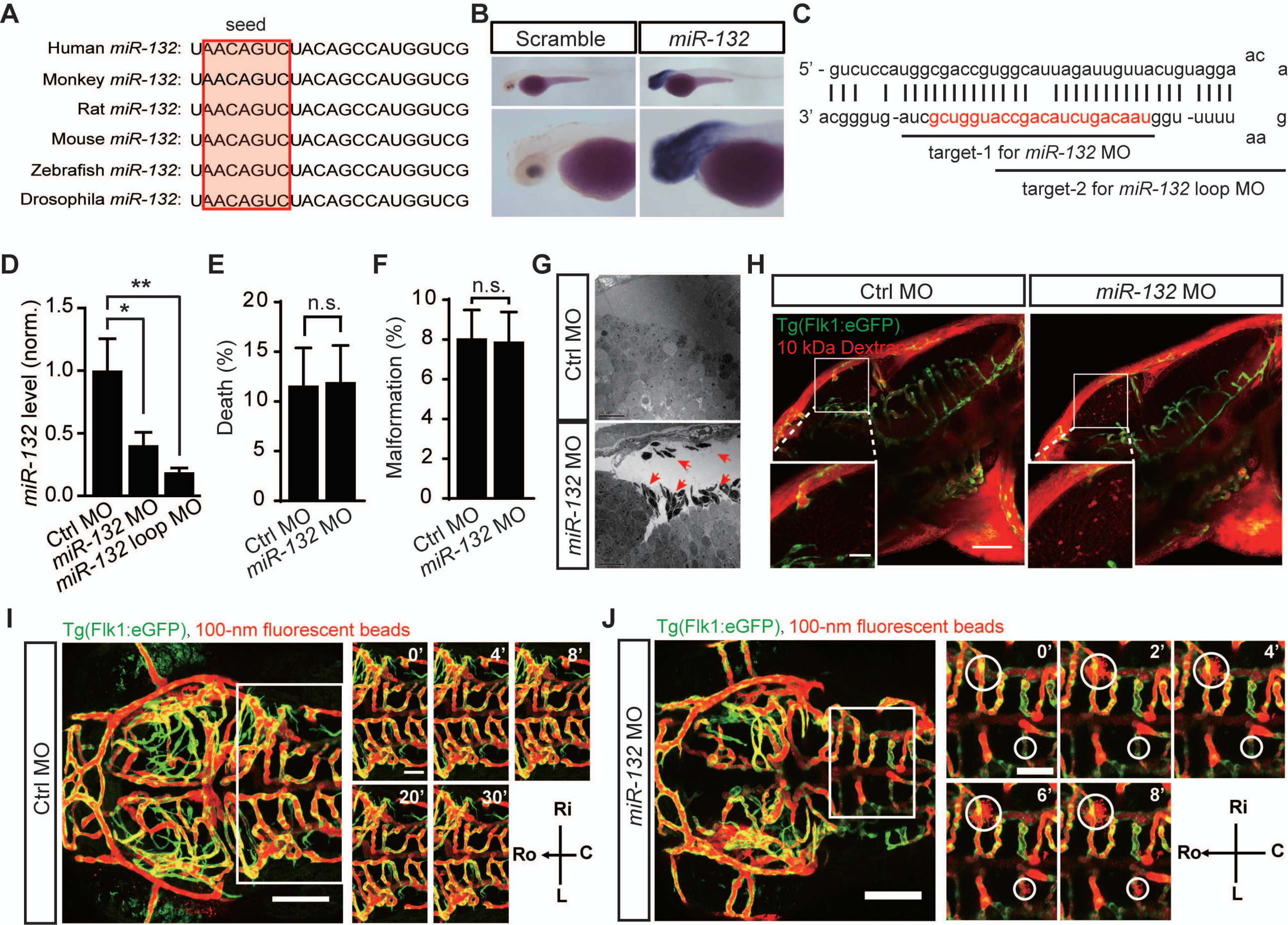

A
seed
Human miR-132: UAACAGUCUACAGCCAUGGUCG
Monkey miR-132: UAACAGUCUACAGCCAUGGUCG
Rat miR-132: UAACAGUCUACAGCCAUGGUCG
Mouse miR-132: UAACAGUCUACAGCCAUGGUCG
Zebrafish miR-132: UAACAGUCUACAGCCAUGGUCG
Drosophila miR-132: UAACAGUCUACAGCCAUGGUCG
B
Scramble
miR-132
C
5' - gucuccauggcgaccguggcauuagauuguuacuguagga
ac
a
3' acgggug -aucgcugguaccgacaucugacaauggu -uuuu
g
aa
target-1 for miR-132 MO
target-2 for miR-132 loop MO
D
miR-132 level (norm.)
Ctrl MO
miR-132 MO
miR-132 loop MO
E
Death (%)
n.s.
Ctrl MO
miR-132 MO
F
Malformation (%)
n.s.
Ctrl MO
miR-132 MO
G
Ctrl MO
miR-132 MO
H
Ctrl MO
miR-132 MO
Tg(Flk1:eGFP)
10 kDa Dextran
I
Tg(Flk1:eGFP), 100-nm fluorescent beads
Ctrl MO
0'
4'
8'
20'
30'
Ri
Ro
C
L
J
Tg(Flk1:eGFP), 100-nm fluorescent beads
miR-132 MO
0'
2'
4'
6'
8'
Ri
Ro
C
L

Supplemental Information, Figure S1. Conserved *miR-132* is necessary for brain vascular integrity. **(A)** Conservation of *miR-132* from *Drosophila* to Human. The red box shows the seed region of *miR-132*. **(B)** Whole-mount *in situ* hybridization showing that *miR-132* is highly expressed in the brain region of zebrafish embryos at 3 days post-fertilization (dpf). **(C)** Schematic showing the design of *miR-132* MO and *miR-132* loop MO. Target-1, *miR-132* MO targeting site; target-2, *miR-132* loop MO targeting site. The sequence of mature *miR-132* is in red. **(D)** Efficiency of MOs in downregulating *miR-132* expression in zebrafish embryos. The experiments were repeated 12 times. **(E)** Death rate of 1-dpf embryos injected with Ctrl MO or *miR-132* MO. The experiments were repeated 7 times. **(F)** Malformation rate of 3-dpf embryos injected wtih Ctrl MO and *miR-132* MO. The experiments were repeated 7 times. **(G)** Representative TEM images of a control embryo and a hemorrhagic *miR-132* morphant showing the accumulation of blood cells (red arrows) in the brain ventricle in *miR-132* morphant. **(H)** Representative projected confocal images showing 10-kDa dextran leakage from brain blood vessels in a 3-dpf Tg(Flk1:eGFP) *miR-132* morphant. The regions outlined by the white rectangles were enlarged. **(I** and **J)** Representative projected confocal time-lapse images showing that red fluorescent bead (100 nm in diameter) leaked from brain blood vessels in a 3-dpf Tg(Flk1:eGFP) control zebrafish **(I)** and *miR-132* morphant **(J)**. In **(J)**, beads leaked from two functional vessels (white circles) which already carried blood flow. Scale bar, 10 μm **(G)**, 100 μm and 25 μm (insert) **(H)**, 100 μm (left) and 50 μm (right) **(I** and **J)**. Error bars, SEM. n.s., no significant; *$P < 0.05$, **$P < 0.01$ (one-way ANOVA with post-hoc Tukey's multiple comparison test for **(D)**; unpaired two-tailed Student's *t* test for **(E)** and **(F)**).
