# Supplementary material for: Neurons secrete miR-132-containing exosomes to regulate brain vascular integrity
Source: Cell Res. 2017 Apr 21;27(7):882–97. doi: 10.1038/cr.2017.62 (PMC5518987; doi:10.1038/cr.2017.62)
Supplement: Supplementary information, Figure S2 — MiR-132 knockdown reduces brain vascular density. [file cr201762x2.pdf]

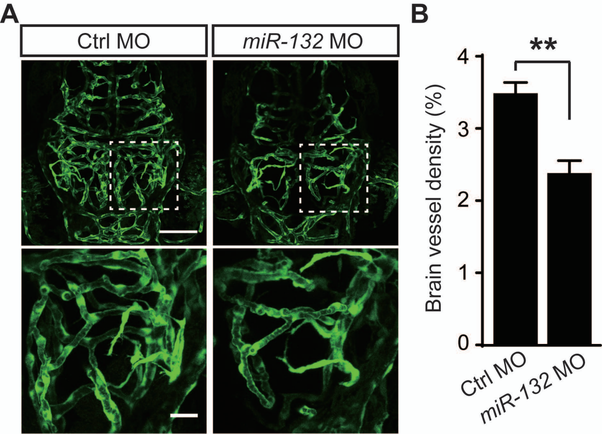
A
Ctrl MO
miR-132 MO
B
**
Brain vessel density (%)
0
1
2
3
4
Ctrl MO
miR-132 MO

Supplementary Information, Figure S2. *MiR-132* knockdown reduces brain vascular density. (A and **B)** Representative projected confocal images **(A)** of 3-dpf Tg(Flk1:eGFP) larvae and summary data **(B)** showing that *miR-132* knockdown led to a decreased brain blood vessel density. 4 control embryos and 11 *miR-132* morphants were analyzed. Scale bars, 100 μm (top) and 25 μm (bottom) **(A)**. Error bars, SEM. $^{**}P < 0.01$ (unpaired two-tailed Student's *t* test for **(B)**).
