# Supplementary material for: Neurons secrete miR-132-containing exosomes to regulate brain vascular integrity
Source: Cell Res. 2017 Apr 21;27(7):882–97. doi: 10.1038/cr.2017.62 (PMC5518987; doi:10.1038/cr.2017.62)
Supplement: Supplementary information, Figure S3 — Impairment of brain vascular integrity in nonhemorrhagic miR-132 morphants. [file cr201762x3.pdf]

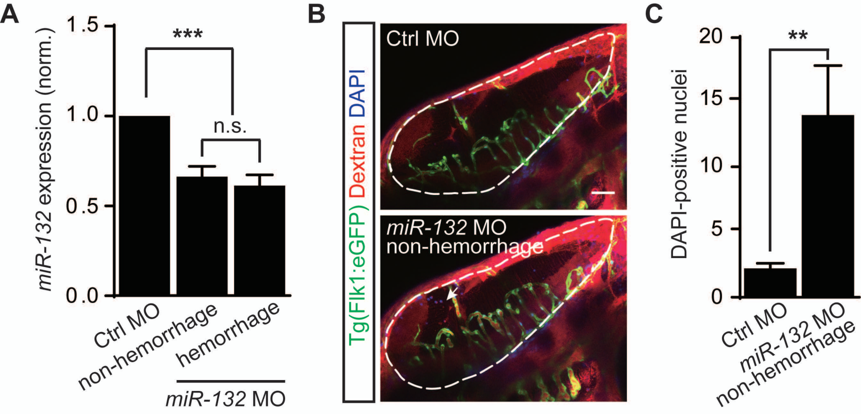
A
B
C
1.5
1.0
0.5
0.0
***
n.s.
miR-132 expression (norm.)
Ctrl MO
non-hemorrhage
hemorrhage
miR-132 MO
Tg(Flk1:eGFP) Dextran DAPI
Ctrl MO
miR-132 MO
non-hemorrhage
20
15
10
5
0
**
DAPI-positive nuclei
Ctrl MO
miR-132 MO
non-hemorrhage

Supplementary Information, Figure S3. Impairment of brain vascular integrity in non-hemorrhagic *miR-132* morphants. (A) Relative expression of *miR-132* in non-hemorrhagic and hemorrhagic *miR-132* morphants. The experiments were repeated for 7 times. **(B** and **C)** Representative projected confocal images **(B)** and summary data **(C)** showing DAPI-positive nuclei (white arrows) in the brain parenchymal of non-hemorrhagic *miR-132* morphants. 7 Ctrl and 7 non-hemorrhagic *miR-132* morphants were analyzed. Scale bar, 50 μm **(B)**. Error bars, SEM. n.s., no significant; $^{**}P < 0.01$, $^{***}P < 0.001$ (one-way ANOVA with post-hoc Tukey's multiple comparison test for **(A)**; unpaired two-tailed Student's *t* test for **(C)**).
