# Supplementary material for: Neurons secrete miR-132-containing exosomes to regulate brain vascular integrity
Source: Cell Res. 2017 Apr 21;27(7):882–97. doi: 10.1038/cr.2017.62 (PMC5518987; doi:10.1038/cr.2017.62)
Supplement: Supplementary information, Figure S4 — Mutation of miR-132 by CRISPR/Cas9. [file cr201762x4.pdf]

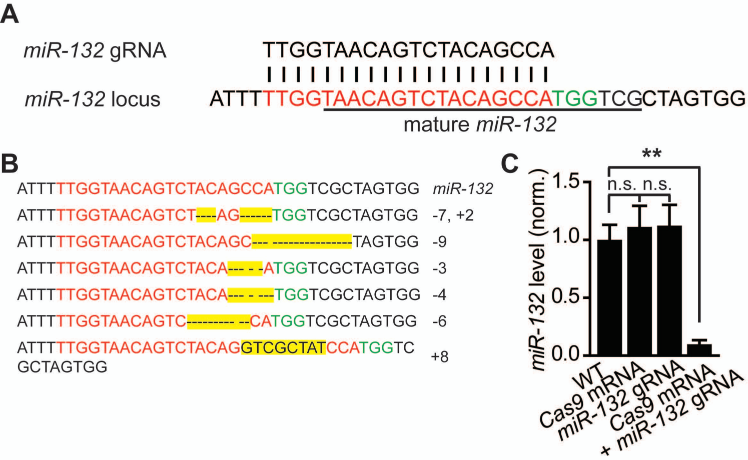
A
miR-132 gRNA
TTGGTAACAGTCTACAGCCA
miR-132 locus
ATTTTTGGTAACAGTCTACAGCCATGGTCGCTAGTGG
mature miR-132
B
ATTTTTGGTAACAGTCTACAGCCATGGTCGCTAGTGG miR-132
ATTTTTGGTAACAGTCT----AG------TGGTCGCTAGTGG -7, +2
ATTTTTGGTAACAGTCTACAGC--- ---------------TAGTGG -9
ATTTTTGGTAACAGTCTACA--- - -ATGGTCGCTAGTGG -3
ATTTTTGGTAACAGTCTACA--- - ---TGGTCGCTAGTGG -4
ATTTTTGGTAACAGTC--------- --CATGGTCGCTAGTGG -6
ATTTTTGGTAACAGTCTACAGGTCGCTATCCATGGTC GCTAGTGG +8
C
**
n.s. n.s.
miR-132 level (norm.)
1.5
1.0
0.5
0.0
WT
Cas9 mRNA
miR-132 gRNA
Cas9 mRNA + miR-132 gRNA

Supplementary Information, Figure S4. Mutation of *miR-132* by CRISPR/Cas9. (A) Design of *miR-132* gRNA. The gRNA targets to the mature sequence of *miR-132* (marked by the line), and the PAM sequence is in green. **(B)** Sequence analysis of *miR-132* mutations caused by co-injection of zebrafish codon-optimized *Cas9* mRNA and *miR-132* gRNA. The red fonts show the target site of *miR-132* gRNA, yellow blanks show mutated sequences, and the green fonts show the PAM sequence. **(C)** Reduced expression of *miR-132* by co-injection of *Cas9* mRNA and *miR-132* gRNA. The experiments were repeated 5 times. Error bars, SEM. n.s., no significant; **$P < 0.01$ (one-way ANOVA with post-hoc Tukey's multiple comparison test for **(C)**).
