# Supplementary material for: Neurons secrete miR-132-containing exosomes to regulate brain vascular integrity
Source: Cell Res. 2017 Apr 21;27(7):882–97. doi: 10.1038/cr.2017.62 (PMC5518987; doi:10.1038/cr.2017.62)
Supplement: Supplementary information, Figure S5 — Cdh5 knockdown impairs brain vascular integrity in larval zebrafish. [file cr201762x5.pdf]

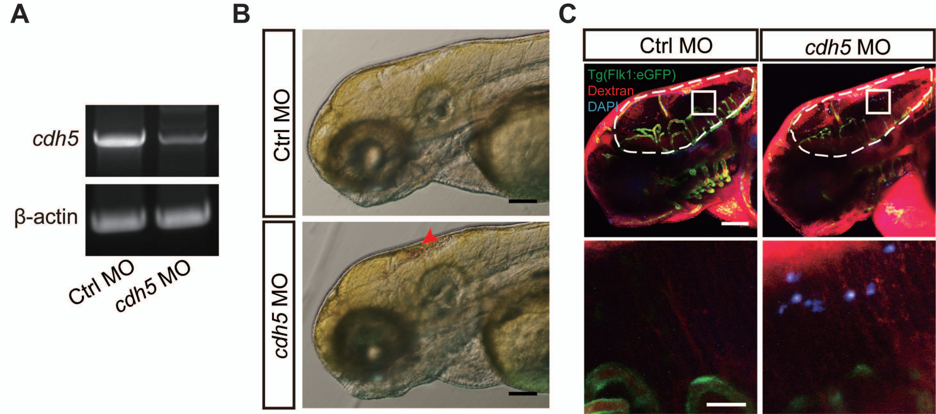
A
cdh5
β-actin
Ctrl MO
cdh5 MO
B
Ctrl MO
cdh5 MO
C
Ctrl MO
cdh5 MO
Tg(Flk1:eGFP)
Dextran
DAPI

Supplementary Information, Figure S5. *Cdh5* knockdown impairs brain vascular integrity in larval zebrafish. (A) RT-PCR analysis showing that *Cdh5* MO significantly reduced the level of *Cdh5* mRNA. **(B** and **C)** Representative images showing that *Cdh5* knockdown caused intracranial hemorrhage (arrowhead in **B**) and DAPI leakage in the brain **(C)**. Scale bars, 100 μm **(B)**, 100 μm (top) and 20 μm (bottom) **(C)**.
