# Supplementary material for: Neurons secrete miR-132-containing exosomes to regulate brain vascular integrity
Source: Cell Res. 2017 Apr 21;27(7):882–97. doi: 10.1038/cr.2017.62 (PMC5518987; doi:10.1038/cr.2017.62)
Supplement: Supplementary information, Figure S6 — Design and efficiency of miR-132 sponge. [file cr201762x6.pdf]

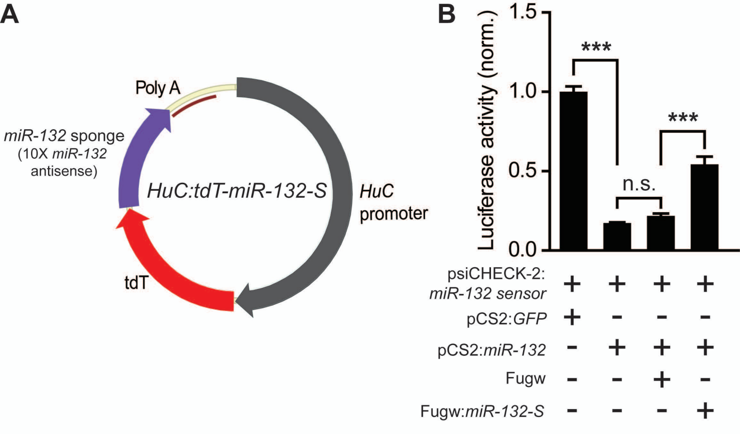
A
Poly A
miR-132 sponge
(10X miR-132
antisense)
HuC:tdT-miR-132-S
HuC
promoter
tdT
B
1.5
1.0
0.5
0.0
Luciferase activity (norm.)
***
n.s.
***
psiCHECK-2:
miR-132 sensor
pCS2:GFP
pCS2:miR-132
Fugw
Fugw:miR-132-S
+ + + +
+ - - -
- + + +
- - + -
- - - +

Supplementary Information, Figure S6. Design and efficiency of *miR-132* sponge. (A) Schematic of *miR-132* sponge (*miR-132-S*), which contained ten repeats of *miR-132* antisense sequence and was inserted into the 3' UTR of *tdTomato* (*tdT*) in the *HuC* vector. **(B)** Luciferase assay in HEK293 cells showing the efficiency of *miR-132-S* in downregulating *miR-132*. The experiments were repeated 3 times. Error bars, SEM. n.s., no significant; ***$P < 0.001$ (one-way ANOVA with post-hoc Tukey's multiple comparison test for **(B)**).
