# Supplementary material for: Neurons secrete miR-132-containing exosomes to regulate brain vascular integrity
Source: Cell Res. 2017 Apr 21;27(7):882–97. doi: 10.1038/cr.2017.62 (PMC5518987; doi:10.1038/cr.2017.62)
Supplement: Supplementary information, Figure S7 — No overlapping between HuC-driven tdTomato expression in neurons and Flk1-driven eGFP expression in ECs. [file cr201762x7.pdf]

Tg(Flk1:eGFP), *HuC:tdT*

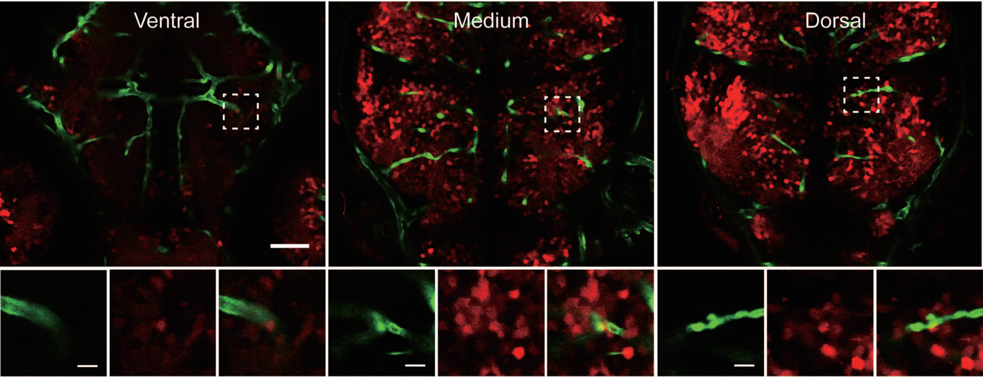

Supplementary Information, Figure S7. No overlapping between *HuC*-driven tdTomato expression in neurons and *Flk1*-driven eGFP expression in ECs. The *HuC:tdT* plasmid was transiently expressed in Tg(Flk1:eGFP) embryos. Representative single slice images at different optical sections showing intensive expression of tdTomato in neuronal somata and fibers, but not in any of eGFP-expressing endothelial cells. Scale bar, 50 μm (top) and 10 μm (bottom).
