# Supplementary material for: Neurons secrete miR-132-containing exosomes to regulate brain vascular integrity
Source: Cell Res. 2017 Apr 21;27(7):882–97. doi: 10.1038/cr.2017.62 (PMC5518987; doi:10.1038/cr.2017.62)
Supplement: Supplementary information, Figure S8 — Internalization of neuronal exosomes into ECs and effect of impaired exosome release on zebrafish intracranial hemorrhage. [file cr201762x8.pdf]

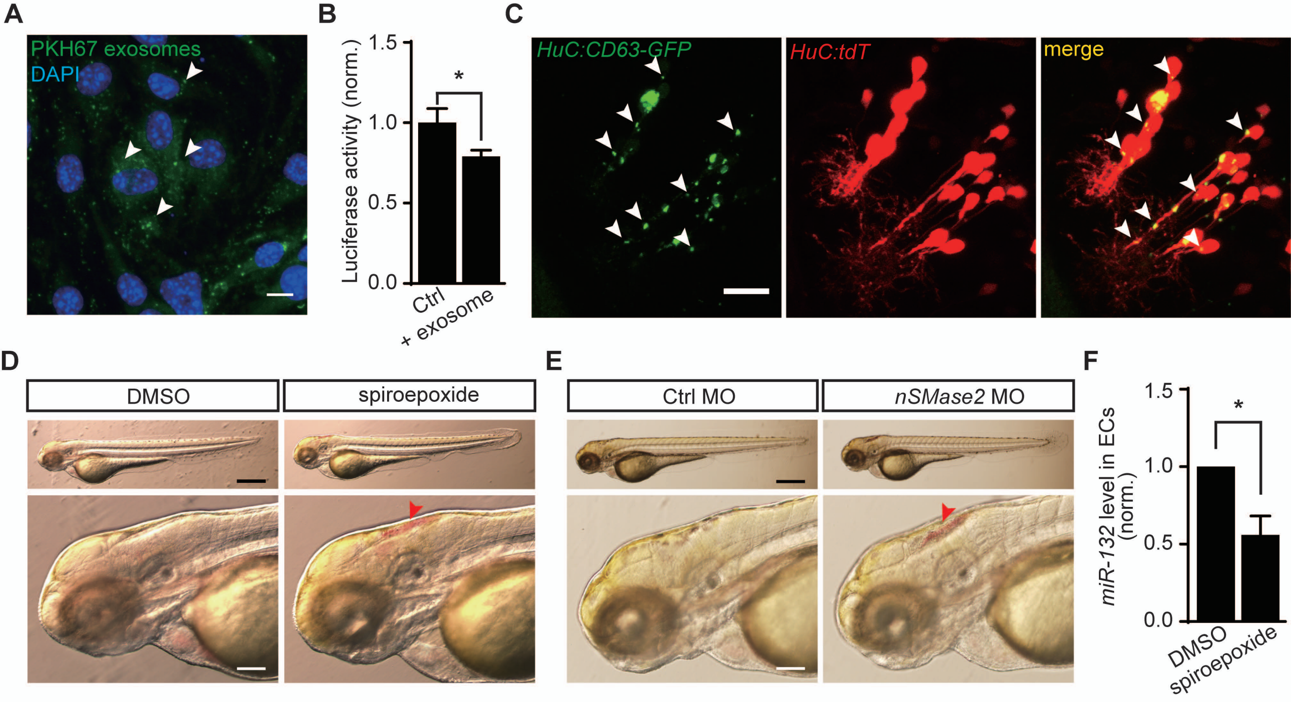
A
PKH67 exosomes
DAPI
B
Luciferase activity (norm.)
1.5
1.0
0.5
0.0
*
Ctrl
+ exosome
C
HuC:CD63-GFP
HuC:tdT
merge
D
DMSO
spiroepoxide
E
Ctrl MO
nSMase2 MO
F
miR-132 level in ECs (norm.)
1.5
1.0
0.5
0.0
*
DMSO
spiroepoxide

Supplementary Information, Figure S8. Internalization of neuronal exosomes into ECs and effect of impaired exosome release on zebrafish intracranial hemorrhage. (A) Neuronal exosomes stained with PKH67 (green dots, arrowheads) were taken up by confluent cultured b.End3 cells. DAPI staining was used for visualizing the nuclei of b.End3 cells. **(B)** Luciferase assay showing that neuronal exosomes (200 ×) repress the luciferase activity of the *miR-132* sensor in cultured b.End3 cells. The experiments were repeated 3 times. **(C)** *HuC*:*CD63-GFP* and *HuC*:*tdT* were co-injected into zebrafish embryos at one-cell stage. The images were taken at 3 dpf. Puncta-like CD63-GFP labeled exosomes (white arrowheads) were observed in neuronal somata and processes. **(D** and **E)** Representative images showing the intracranial hemorrhage (arrowheads) effect of nSMase2 blockade with spiroepoxide **(D)** or *nSMase2* knockdown with MO **(E)** in 3-dpf zebrafish larvae. **(F)** Reduced expression of *miR-132* in ECs by spiroepoxide treatment. Scale bar, 10 μm **(A)**, 20 μm **(C)**, 400 μm (top) and 100 μm (bottom) **(D** and **E)**. Error bars, SEM. *$P < 0.05$ (unpaired two-tailed Student's *t* test for **(B)** and **(F)**).
