# Supplementary material for: Neurons secrete miR-132-containing exosomes to regulate brain vascular integrity
Source: Cell Res. 2017 Apr 21;27(7):882–97. doi: 10.1038/cr.2017.62 (PMC5518987; doi:10.1038/cr.2017.62)
Supplement: Supplementary information, Figure S9 — Prediction of the target genes of miR-132 in zebrafish. [file cr201762x9.pdf]

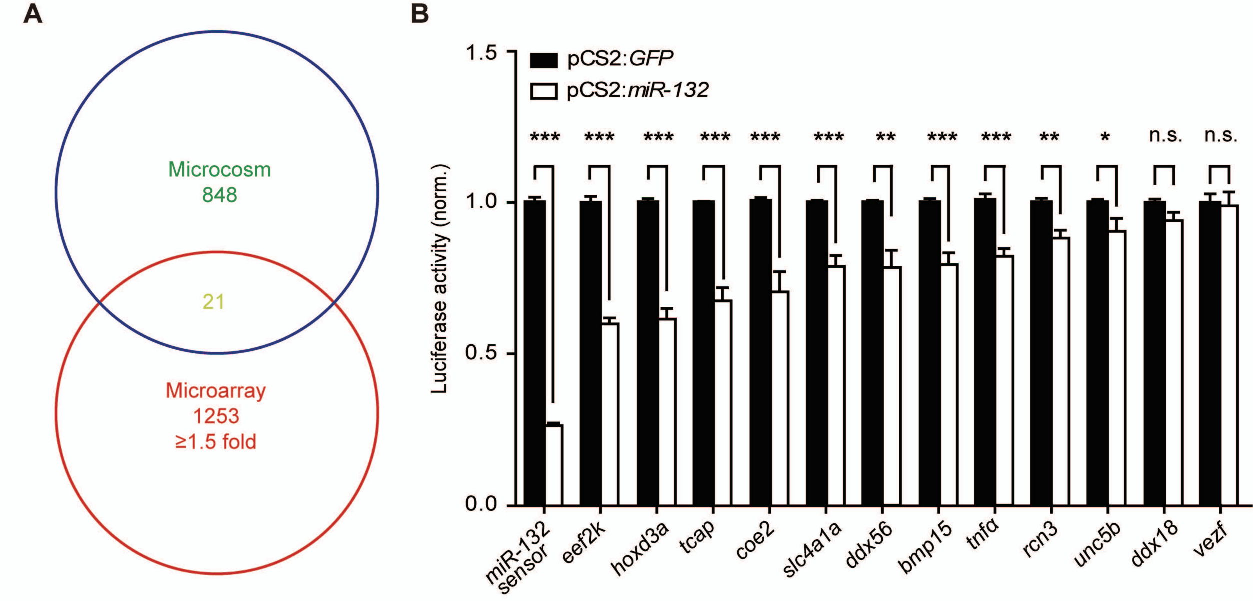

A
Microcosm
848
21
Microarray
1253
≥1.5 fold
B
pCS2:GFP
pCS2:miR-132
Luciferase activity (norm.)
1.5
1.0
0.5
0.0
***
***
***
***
***
***
**
***
***
**
*
n.s.
n.s.
miR-132 sensor
eef2k
hoxd3a
tcap
coe2
slc4a1a
ddx56
bmp15
tnfα
rcn3
unc5b
ddx18
vezf

Supplementary Information, Figure S9. Prediction of the target genes of *miR-132* in zebrafish. (A) 21 candidate genes were got after combination of the microarray data and the predicted targets by the Microcosm algorithm. **(B)** Luciferase assay in HEK293 cells showing the repressing efficiency of *miR-132* on 12 annotated candidate genes. The experiments were repeated 3 times. Error bars, SEM. n.s., no significant; *$P < 0.05$, **$P < 0.01$, ***$P < 0.001$ (unpaired two-tailed Student's t test for **(B)**).
