# Supplementary material for: Neurons secrete miR-132-containing exosomes to regulate brain vascular integrity
Source: Cell Res. 2017 Apr 21;27(7):882–97. doi: 10.1038/cr.2017.62 (PMC5518987; doi:10.1038/cr.2017.62)
Supplement: Supplementary information, Figure S10 — Role of eef2k in mediating miR-132 effects on brain vascular integrity. [file cr201762x10.pdf]

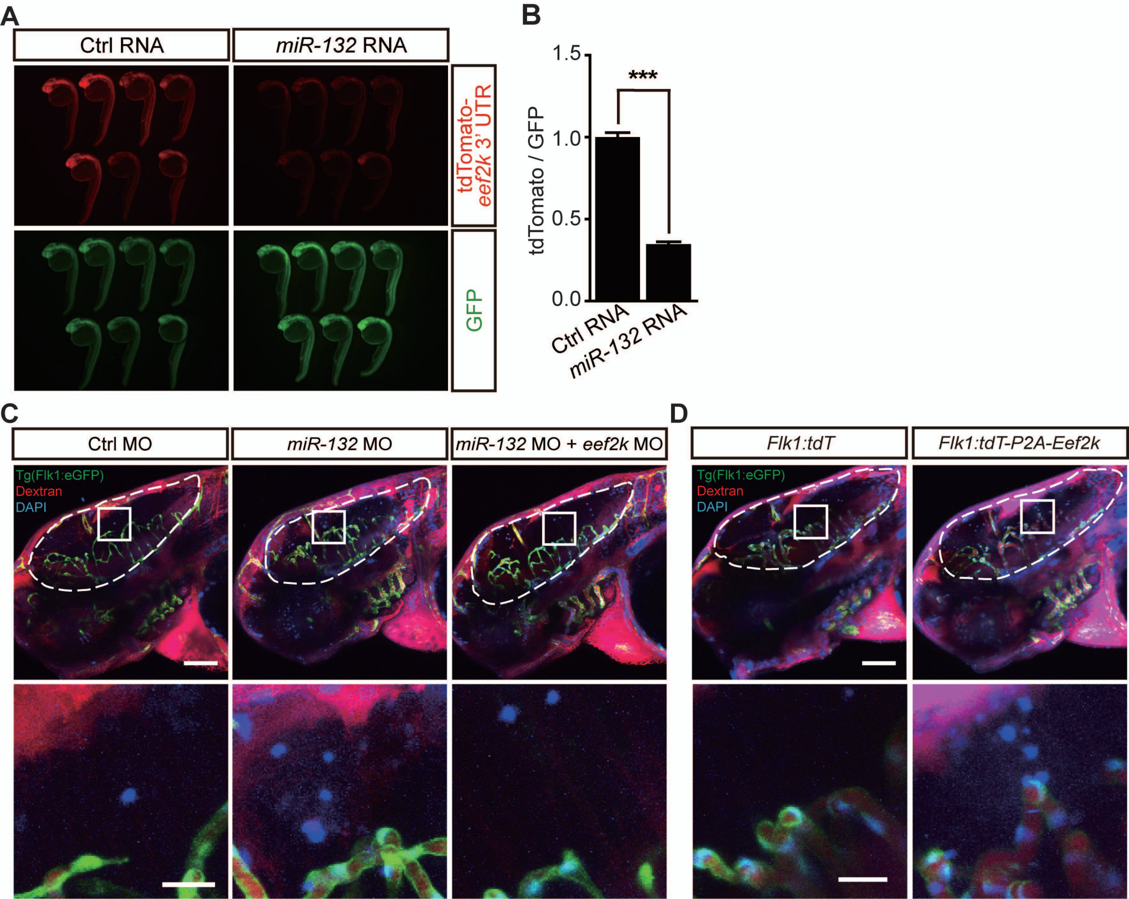
A
Ctrl RNA
miR-132 RNA
tdTomato-eef2k 3' UTR
GFP
B
tdTomato / GFP
1.5
1.0
0.5
0.0
***
Ctrl RNA
miR-132 RNA
C
Ctrl MO
miR-132 MO
miR-132 MO + eef2k MO
Tg(Flk1:eGFP)
Dextran
DAPI
D
Flk1:tdT
Flk1:tdT-P2A-Eef2k
Tg(Flk1:eGFP)
Dextran
DAPI

Supplementary Information, Figure S10. Role of *eef2k* in mediating *miR-132* effects on brain vascular integrity. (A and **B)** Representative images **(A)** and Summary **(B)** showing the injection of *miR-132* RNA together with *tdTomato-eef2k* 3' UTR mRNA and *GFP* mRNA reduces the expression of tdTomato in zebrafish embryos at 1 dpf. GFP signal is used as an internal control. **(C)** Representative images showing the rescue effect of *eef2k* knockdown on *miR-132* knockdown-induced DAPI leakage in the brain. **(D)** Representative images showing the effect of EC-specific *Eef2k* overexpression on DAPI leakage in the brain. Scale bars, 100 μm (top) and 20 μm (bottom) **(C** and **D)**. Error bars, SEM. ***$P < 0.001$ (unpaired two-tailed Student's t test).
